# Dopamine D1/D5 Receptor Signaling Is Involved in Arrhythmogenesis in the Setting of Takotsubo Cardiomyopathy

**DOI:** 10.3389/fcvm.2021.777463

**Published:** 2022-02-04

**Authors:** Mengying Huang, Zhen Yang, Yingrui Li, Huan Lan, Lukas Cyganek, Goekhan Yuecel, Siegfried Lang, Karen Bieback, Ibrahim El-Battrawy, Xiaobo Zhou, Martin Borggrefe, Ibrahim Akin

**Affiliations:** ^1^First Department of Medicine, Medical Faculty Mannheim, University Medical Centre Mannheim (UMM), University of Heidelberg, Mannheim, Germany; ^2^Key Laboratory of Medical Electrophysiology of Ministry of Education and Medical Electrophysiological Key Laboratory of Sichuan Province, Institute of Cardiovascular Research, Southwest Medical University, Luzhou, China; ^3^DZHK (German Center for Cardiovascular Research), Partner Site, Göttingen, Germany; ^4^Stem Cell Unit, Clinic for Cardiology and Pneumology, University Medical Center Göttingen, Göttingen, Germany; ^5^DZHK (German Center for Cardiovascular Research), Partner Site, Heidelberg, Germany; ^6^DZHK (German Center for Cardiovascular Research), Partner Site, Mannheim, Germany; ^7^Institute of Transfusion Medicine and Immunology, University Medical Centre Mannheim (UMM), University of Heidelberg, Mannheim, Germany

**Keywords:** Takotsubo cardiomyopathy, arrhythmia, catecholamine excess, D1/D5 dopamine receptor, human-induced pluripotent stem cell-derived cardiomyocytes

## Abstract

**Background:**

Previous studies suggested involvement of non-ß-adrenoceptors in the pathogenesis of Takotsubo cardiomyopathy (TTC). This study was designed to explore possible roles and underlying mechanisms of dopamine D1/D5 receptor coupled signaling in arrhythmogenesis of TTC.

**Methods:**

Human-induced pluripotent stem cell-derived cardiomyocytes (hiPSC-CMs) were challenged by toxic concentration of epinephrine (Epi, 0.5 mM for 1 h) for mimicking the catecholamine excess in setting of TTC. Specific receptor blockers and activators were used to unveil roles of D1/D5 receptors. Patch clamp, qPCR, and FACS analyses were performed in the study.

**Results:**

High concentration Epi and two dopamine D1/D5 receptor agonists [(±)-SKF 38393 and fenoldopam] reduced the depolarization velocity and prolonged the duration of action potentials (APs) and caused arrhythmic events in iPSC-CMs, suggesting involvement of dopamine D1/D5 receptor signaling in arrhythmogenesis associated with QT interval prolongation in the setting of TTC. (±)-SKF 38393 and fenoldopam enhanced the reactive oxygen species (ROS)-production. H_2_O_2_ (100 μM) recapitulated the effects of (±)-SKF 38393 and fenoldopam on APs and a ROS-blocker *N*-acetylcysteine (NAC, 1 mM) abolished the effects, suggesting that the ROS-signaling is involved in the dopamine D1/D5 receptor actions. A NADPH oxidases blocker and a PKA- or PKC-blocker suppressed the effects of the dopamine receptor agonist, implying that PKA, NADPH oxidases and PKC participated in dopamine D1/D5 receptor signaling. The abnormal APs resulted from dopamine D1/D5 receptor activation-induced dysfunctions of ion channels including the Na^+^ and L-type Ca^2+^ and I_Kr_ channels.

**Conclusions:**

Dopamine D1/D5 receptor signaling plays important roles for arrhythmogenesis of TTC. Dopamine D1/D5 receptor signaling in cardiomyocytes might be a potential target for treating arrhythmias in patients with TTC.

## Introduction

Takotsubo cardiomyopathy (TTC) was initially described ~25 years ago (1). The main feature of the disease is temporary and reversible systolic abnormalities in the apex of the left ventricle and/or involvement of right ventricle with clinical manifestations similar to myocardial infarction (2, 3). The most common symptoms of patients with TTC are chest pain and dyspnea. In severe cases, patients suffer from cardiac arrest, cardiogenic shock and ventricular arrhythmia (4). About 90% of patients with TTC are postmenopausal women who experience multiple stresses before the onset (3, 5). Although several possible causes have been proposed, such as catecholamine cardiotoxicity, metabolic disorders, coronary microvascular injury, multivessel epicardial coronary artery spasm and thyroidal dysfunction, the pathophysiology of TTC has not been fully established (6). A commonly accepted hypothesis is the surge of catecholamines caused by intense psychological or physical stress (4, 7).

So far, the TTC model has been successfully simulated on animal and human induced pluripotent stem cell-derived cardiomyocytes (hiPSC-CMs) by using high concentrations of catecholamines and demonstrated that both beta and alpha adrenoceptors related signaling are important for the pathogenesis of TTC (8–11). However, some clinical reports indicate that the plasma dopamine level in patients with stress cardiomyopathy is significantly increased (12–14). Moreover, high concentration of dopamine successfully induced TTC-like cardiac dysfunction in animal model (15). More importantly, studies showed that some TTC-patients did not profit from use of ß-blocker (16–18). All of these evidences suggest that dopamine-receptor activation may contribute to the pathogenesis of TTC.

Dopamine receptors are a subclass of the super family of G protein-coupled receptors and consisting of two families (19). The D1 family include of D1- and D5-receptor subtypes and the D2 family include of D2-, D3-, and D4-receptor subtypes, which have been identified in a number of organs and tissues in human (20). Among them, D1, D2, D4, and D5 are found in the native human heart (21), but only D1-like receptors (D1 and D5) exist in endocardium that are linked to adenylyl-cyclase stimulation, which can mediate the functional activity of Ca^2+^, K^+^, Na^+^ channels by regulating G protein coupling (19, 21–23). However, in human cardiomyocytes, studies on the potential mechanism of ion channel dysfunction and arrhythmias caused by dopamine receptor activation in TTC have not been conducted. Therefore, we designed the current study to assess the importance of dopamine D1/D5 receptor related signaling for the pathogenesis of TTC, focusing on the mechanisms of arrhythmias.

## Materials and Methods

### Generation of Human iPS Cells

The human-induced pluripotent stem cells (hiPSCs) from three male healthy donors were created by integration-free methods under feeder-free culture conditions, as descripted previously (24). In brief, the hiPSC line UMGi014-B clone 1 (ipWT1.1) was generated from dermal fibroblasts by using non-integrated episomal 4-in-1 CoMiP reprogramming plasmids (Addgene, #63726), hiPSC line UMGi124-A clone 11 (isVHFx-R1) was generated from dermal fibroblasts by integration-free Sendai virus, and hiPSC line UMGi130-A clone 5 (isWT11.5) was generated from peripheral mononuclear blood cells by integration-free Sendai virus.

### Generation of hiPSC Derived Cardiomyocytes (hiPSC-CMs)

In our previous studies, the generation of hiPSC-CMs has been described (25). Briefly, culture dishes and wells coated with matrigel (Corning, Kaiserslautern, Germany) as well as the culture medium TeSR-E8 (Stemcell Technologies, Köln, Germany) were used for culturing hiPSCs. For culturing hiPSC-CMs, the medium RPMI 1640 Glutamax (Life Technologies, Darmstadt, Germany) containing sodium pyruvate, penicillin/streptomycin, B27 (Life Technologies) and ascorbic acid (Sigma Aldrich, Taufkirchen, Germany) was used. In the first 2 weeks, CHIR99021 (MiltenyiBiotec, Bergisch Gladbach, Germany), BMP-4 (R&D Systems, Wiesbaden, Germany), Activin A (R&D Systems), FGF-2 (MiltenyiBiotec), and IWP-4 (MiltenyiBiotec) were used at several times for inducing the cells to be differentiated into hiPSC-CMs. In the 3rd week, a cardiomyocyte-selection medium containing sodium lactate (Sigma Aldrich) and RPMI-medium without glucose and glutamine (Biological Industries, Cromwell, USA) was used for 4–7 days (usually 5 days) for selecting cardiomyocytes. Then, the survived cardiomyocytes were cultured in cardiac medium. At 40–60 days of differentiation, hiPSC-CMs were used for different experiments. To confirm the successful differentiation of hiPSC-CMs, the expression of different cardiac markers was examined as shown in our recent study (26). The differentiation was performed every 3–4 weeks, cells from different differentiations were measured and data were combined.

### Quantitative Polymerase-Chain-Reaction Assays

RNA was reverse transcribed and converted to cDNA with oligo (dT)_15_ primers with AMV reverse transcriptase following the protocol. Relative mRNA level was calculated as: the mRNA level of the gene of interest relative to that of the housekeeping gene GAPDH in samples from treated or untreated (Control) cells was obtained from the ΔΔCT method, based on the threshold cycle (CT), as fold change = 2^−Δ(Δ*CT*)^, where ΔCT = CT_geneofinterest_ – CT_GAPDH_ and Δ(ΔCT) = ΔCT_treated_ –ΔCT _Control_ (27). For each experimental group, the cDNA amount of three or more cell culture wells were examined as biological replicates. The sample of each cell culture well was measured two times as technical replicates. The information of used primers is provided in Supplementary Table 1.

### Fluorescence-Activated Cell Sorting (FACS)

The ROS generation of hiPSC-CM was assessed with 2′,7′-Dichlorofluorescin diacetate (DCFH-DA, sigma) technique following the ROS assay kit instructions. DCFH-DA is a non-polar fluorescence probe that can enter a cell, and in the cell, it is changed into DCFH and can be detected by flow cytometry. The hiPSC-CMs were detached by 0.05% Trypsin-EDTA (Life Technologies) for 2–4 min at 37°C. Thereafter, RPMI medium containing 10% FBS was added. Then, a centrifugation of 250 × *g* was performed for 4 min at room temperature. Next, the supernatant was taken away and the hiPSC-CMs were resuspended in basic culture medium. The hiPSC-CMs were transferred into the 15 ml tubes with 1 × 10^6^ cells/tube and incubated with 10 μM DCFH-DA at 37°C for 30 min in the dark. The hiPSC-CMs were then washed thrice by PBS and measured by BD FACSCanto™ II (Becton Dickinson, Heidelberg, Germany). Analyses were conducted with a quantitative method *via* BD FACS Diva software (Version 8.0.1). More than 25,000 events were sampled and analyzed per experimental condition.

### Immunofluorescence (IF) Staining

The hiPSC-CMs were detached by 0.05% Trypsin-EDTA (Life Technologies) for 2–4 min at 37°C. The RPMI medium containing 10% FBS was added. The hiPSC-CMs were then centrifuged at 250 × *g* for 4 min at room temperature. Thereafter, the supernatant was taken away and the hiPSC-CMs were resuspended in basic culture medium and pipetted onto culture slides (FALCON 354114). The slides stayed at room temperature for overnight. On next day, hiPSC-CMs were washed by PBS thrice, and then fixed by 4% Paraformaldehyde (Sigma) at room temperature for 20 min. Then, cells were washed thrice by PBS and were permeabilized by 0.1% Triton-X100 (Carl Roth) for 10 min. Next, hiPSC-CMs were washed trice by PBS and blocked by 5% bovine serum albumin (BSA; Sigma-Aldrich) in PBS at 4°C for 1 h. Primary antibodies were incubated at 4°C in 5% BSA overnight. Then, hiPSC-CMs were washed twice by PBS and incubated with secondary antibodies conjugated to Alexa Fluor 488 or 642 (1:200) at room temperature for 1 h. Finally, hiPSC-CMs were washed twice by PBS and incubated with DAPI (Biozol) at room temperature for 10 min in the dark. Images were analyzed by the Confocal Microscope TCS SP-8 upright (Leica, Germany) with Plan-Apochromat 40 × /0.6 objective.

The information of antibodies used in the study is provided in Supplementary Table 2.

### Patch-Clamp

For patch-clamp measurements, cardiomyocytes at 40–60 days of differentiation were dissociated from 6 well plates by collagenase type I and plated on matrigel-coated 3.5 cm petri dishes as single cells. The patch-clamp whole-cell recording technique was employed to measure the action potential (AP) and channel currents at RT (22–24°C). Pipette resistance ranged from 1 to 2 MΩ and 4 to 5 MΩ for current and AP measurements, respectively. The electrode offset potential was zero-adjusted before a Giga-seal was formed. When a Giga-seal was formed, the fast capacitance was first compensated and then the membrane under the pipette tip was broken by negative pressure to obtain the whole-cell configuration. The signal was sampled at 10 kHz and filtered at 2 kHz using the EPC10 Patch-master digitizer hardware (HEKA Germany) and Fit-master software (HEKA Germany). The action potential was measured by current clamp mode. The spontaneous AP was measured in CC-mode in spontaneously beating hiPSC-CMs. For measuring the APs at a fixed frequency, brief current pulses (2 ms, 1 nA) of 1 Hz were employed for evoking APs. The junction potential (4–6 mV) in AP recordings was not corrected. Recordings of currents and paced APs were started when currents and APs became stable. The spontaneous AP was recorded within the first 100 s after a whole-cell configuration was obtained since after 100 s the spontaneous APs may terminate. To analyze the arrhythmic events, all the events including normal APs, early afterdepolarization (EAD)-like events, which were defined as depolarizations in repolarization phase (phase 3) of AP, delayed afterdepolarization (DAD)-like events, which were defined as under-threshold depolarizations in phase 4 of APs, and triggered activity, which was defined as APs triggered by abnormal over-threshold triggers/stimulations, were counted in control and drug-treated hiPSC-CMs. The number of every type of events in each group was divided by the number of all events (normal and arrhythmic events) to obtain the percentage of arrhythmic events. The number of cells showing arrhythmic evens either EAD-like or DAD-like or triggered activity was divided by the total number of counted cells in a group to obtain the ratio of cells showing arrhythmic events (Table 1).

**Table 1:** Analysis of arrhythmic events relating to dopamine receptors.

**Group**	**Control**	**Epi**	**Epi+SCH23390**	**SKF 38393**	**Fenoldopam**
Cell number	12	12	12	12	12
Number of cells showing arrhythmia	6	12	9	12	12
Beating rate (bpm)	26.1 ± 3.7	20.5 ± 1.7	23.9 ± 1.8	35.1 ± 2.2	46.8 ± 5.8^**^
Arrhythmic events (%)	8.1% ± 3.1%	26.5 ± 2.1^**^	9.3 ± 2.5	26.3% ± 4%^**^	29.7% ± 4.4%^**^
Arrhythmia counts	20	65	27	102	144
Number of DAD-like events	16	35	14	65	88
Number of EAD-like events	2	20	9	15	23
Number of triggered activities	2	10	4	22	33
Normal rhythm counts	258	181	254	248	417

** *p < 0.01. The p-values vs. control were determined by one-way ANOVA with Holm-Sidak post-test*.

The bath solution (PSS) for AP measurements consists of (mmol/l): 130 NaCl, 5.9 KCl, 2.4 CaCl_2_, 1.2 MgCl_2_, 11 glucose, 10 HEPES, pH 7.4 (NaOH). The pipette solution contains (mmol/l): 10 HEPES, 126 KCl, 6 NaCl, 1.2 MgCl_2_, 5 EGTA, 11 glucose, and 1 MgATP, pH 7.2 (KOH).

The bath solution for peak sodium current recording consists of 20 mmol/L NaCl, 130 mmol/L CsCl, 1.8 mmol/L CaCl_2_, 1 mmol/L MgCl_2_, 10 mmol/L HEPES, 10 mmol/L glucose, and 0.001 mmol/L nifedipine. The pH value was adjusted to 7.4 with CsOH. The pipette solution for peak sodium current consists of 10 mmol/L NaCl, 135 mmol/L CsCl, 2 mmol/L CaCl_2_, 3 mmol/L Mg-ATP, 5 mmol/L EGTA, 10 mmol/L HEPES, and pH 7.2 (CsOH).

The extracellular solution for measuring L-type (I_Ca−L_) calcium channel current consists of (mmol/l): 140 TEA-Cl, 5 CaCl_2_, 1 MgCl_2_, 0.001 E-4031, 10 HEPES, 0.02 TTX, 3 4-AP, pH 7.4 (CsOH). The intracellular solution consists of (mmol/l): 6 NaCl, 135 CsCl, 2 CaCl_2_, 3 Mg ATP, 2 TEA-Cl, 5 EGTA, 10 HEPES, and pH7.2 (CsOH).

To isolate I_Kr_ from other K^+^ channel currents, the Cs^+^ currents conducted by I_Kr_ channels were measured. The extracellular and intracellular solution for the Cs^+^ current measurement consists of (mmol/L): 140 CsCl, 2 MgCl_2_, 10 HEPES, 10 Glucose, and pH = 7.4 (CsOH).

The information of drugs and reagents is provided in the Supplementary Table 3.

### Statistics

Data are shown as means ± SEM and were analyzed using InStat^©^ (GraphPad, San Diego, CA, USA) and SigmaPlot 11.0 (Systat GmbH, Erkrath Germany). To decide whether parametric or non-parametric tests were to be used for analysis, the Kolmogorov Smirnov test was first performed. Outliers in data were excluded in analyses. To determine outliers, the first quantile (Q1), the third quantile (Q3) and the interquartile range (IQR) were calculated by Software Excel. The lower limit and upper limit were defined as Q1-(1.5^*^IQR) and Q3+(1.5^*^IQR), respectively. All the data below the lower limit or over the upper limit were defined as outliers. Student's *t*-test was used to compare continuous variables with normal distributions, while the Mann–Whitney *U*-test was used to compare continuous variables with non-normal distributions. For comparing categorical variables, the Fisher-test was used. For comparing parametric data of more than two groups, one-way ANOVA with a Holm-Sidak post-test for multiple comparisons was carried out. An unpaired Student's *t*-test was performed for comparing two independent groups with normal distributions. *p* < 0.05 (two-tailed) was considered significant.

## Results

### The Dopamine D1/D5 Receptor Activation Contributes to Occurrence of Arrhythmic Events Induced by High-Concentration of Epinephrine

**Figure 1:**
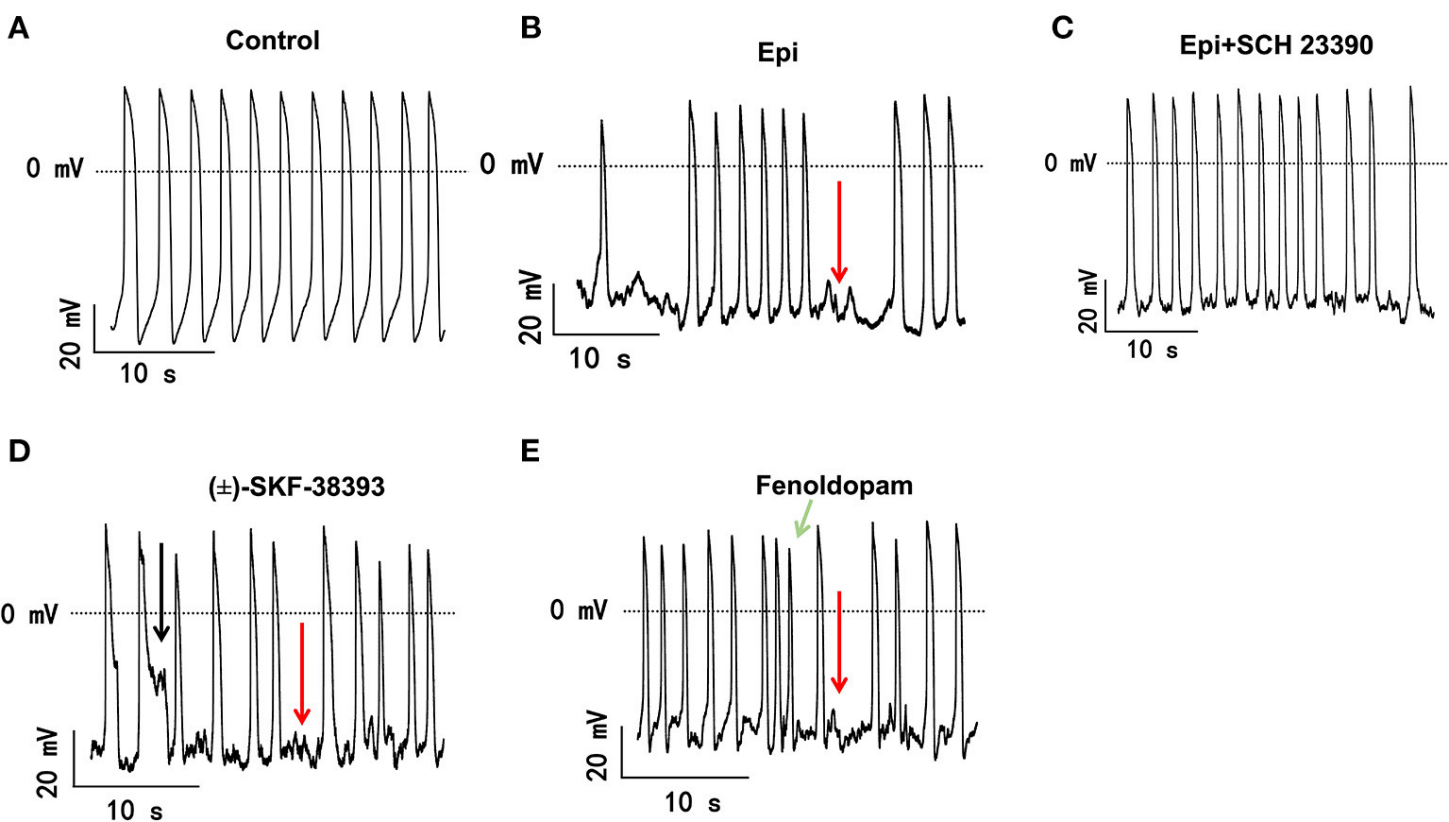
The dopamine D1/D5 receptor activation contributes to occurrence of arrhythmic events. hiPSC-CMs were treated by vehicle (Control) or 500 μM Epi or Epi plus 10 μM SCH 23390 or 50 μM (±)-SKF 38393 or 5 μM fenoldopam for 1 h. Spontaneous APs of spontaneously beating hiPSC-CMs were measured in the first 100 s after the whole cell configuration was obtained. The arrhythmic events including EAD-like (black arrows) or DAD-like and failed AP (red arrows) events or triggered activity (green arrows) in each cell were counted and divided by total events to obtain the percentage of arrhythmic events. Data were summarized in Table 1. **(A)** Representative traces of spontaneous APs recorded in a cell of control group. **(B)** Representative AP traces in a cell of Epi-group. **(C)** Representative AP traces in a cell of Epi+SCH 23390 group. **(D)** Representative AP traces in a cell of (±)-SKF 38393 group. **(E)** Representative AP traces in a cell of fenoldopam group.

The study used hiPSC-CMs from three healthy donors. Our recent publications confirmed the successful differentiation of hiPSCs into hiPSC-CMs by using qPCR, immunofluorescence and FACS analysis regarding the expression of cardiac markers (26). In this study, the cardiac marker cTnT (TNNT2) was also shown by immunofluorescence (Supplementary Figure 1B) and FACS (Figures 5A,B) analyses. It has been shown that dopamine receptors exist in cardiomyocytes (21). We next examined the mRNA expression levels of dopamine receptors in our hiPSC-CMs by qPCR (Supplementary Figure 1A). We observed that DRD1 and DRD5 are highly expressed in hiPSC-CMs. Furthermore, the immunostaining analysis confirmed the expression of D1 dopamine receptor in hiPSC-CMs (Supplementary Figure 1B). It has been proven that high concentrations of epinephrine can cause arrhythmia (11). In order to verify whether dopamine receptor activation could also be involved in the occurrence of arrhythmia, we first treated the spontaneously beating cells with Epi (500 μM) in the presence or absence of 10 μM SCH 23390 (a dopamine D1/D5 receptor blocker). The result showed that SCH 23390 prevented the Epi-induced arrhythmia events (Figures 1A–C). Therefore, two kinds of dopamine D1/D5 receptor-specific agonists (±)-SKF 38393 (50 μM) and fenoldopam (5 μM) were used to spontaneously beating cells and arrhythmic events including EAD- (early afterdepolarization) like, DAD- (delayed afterdepolarization) like events, failed APs and triggered activity were analyzed. The results showed that (±)-SKF 38393 and fenoldopam significantly increased the occurrence of arrhythmic events (Figures 1A,D,E; Table 1). In addition, both (±)-SKF 38393 and fenoldopam increased the cell beating, especially fenoldopam showed a significant effect (Table 1). In the presence of (±)-SKF 38393 and fenoldopam, more cells showed arrhythmic events (Table 1).

### The Dopamine D1/D5 Receptor Activation Is Involved in Toxic Effects of Epinephrine on Action Potentials

**Figure 2:**
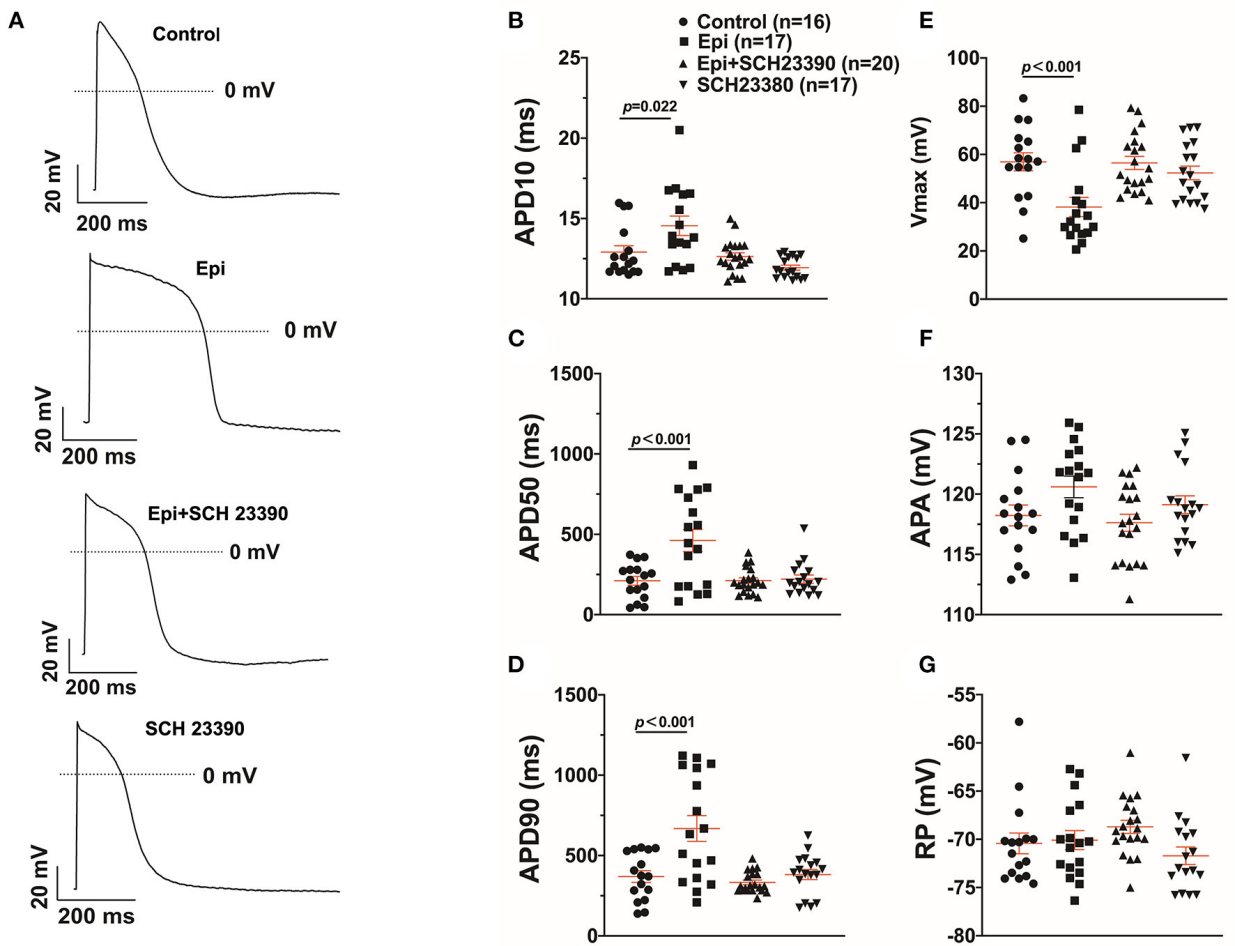
Dopamine D1/D5 receptor signaling contributed to effect of epinephrine on action potential. hiPSC-CMs were challenged by either vehicle (Control) or 500 μM epinephrine (Epi) or epinephrine plus 10 μM SCH 23390 or SCH 23390 alone for 1 h. Action potentials (APs) were recorded at 1 Hz. **(A)** Examples of APs in a cell of each group. **(B)** Mean values of APD10 in each group cells. **(C)** Mean values of APD50 in each group cells. **(D)** Mean values of APD90 in each group cells. **(E)** Mean values of Vmax of APs in each group cells. **(F)** Mean values of AP amplitude (APA) in each group cells. **(G)** Mean values of resting potential (RP) in each group cells. “*n*” numbers in **(B)** are for **(B–G)**. The *p*-values vs. Control were decided by the analysis of one-way ANOVA with Holm-Sidak post-test.

Arrhythmias are usually related to changes of action potentials (APs). So, APs were measured in hiPSC-CMs challenged by a toxic concentration of epinephrine (Epi, 500 μM) for 1 h in the absence or presence of a dopamine D1/D5 receptor blocker (SCH 23390, 10 μM). The results showed that Epi prolonged the duration of action potential at 10% repolarization (APD10) from 12.9 ± 0.40 to 14.55 ± 0.60 ms, the duration of action potential at 50% repolarization (APD50) from 210.86 ± 27.40 to 461.22 ± 68.22 ms and the duration of action potential duration at 90% repolarization (APD90) from 369.15 ± 36.58 to 667.98 ± 80.18 ms, respectively (Figures 2A–D). Interestingly, the dopamine D1/D5 receptors blocker prevented the Epi-effect on action potentials (Figures 2A–D). The dopamine D1/D5 receptor blocker alone showed no effect on APDs (Figures 2A–D). The maximal depolarization velocity (Vmax) of APs was attenuated by Epi and the Epi-effect was reversed by SCH 23390 (Figure 2E). Epi or the dopamine D1/D5 receptor blocker alone showed no effect on the resting potential (RP) and the AP amplitude (APA) (Figures 2F,G). These data indicate that dopamine D1/D5 receptor activation plays a key role in Epi-induced effects on APs.

**Figure 3:**
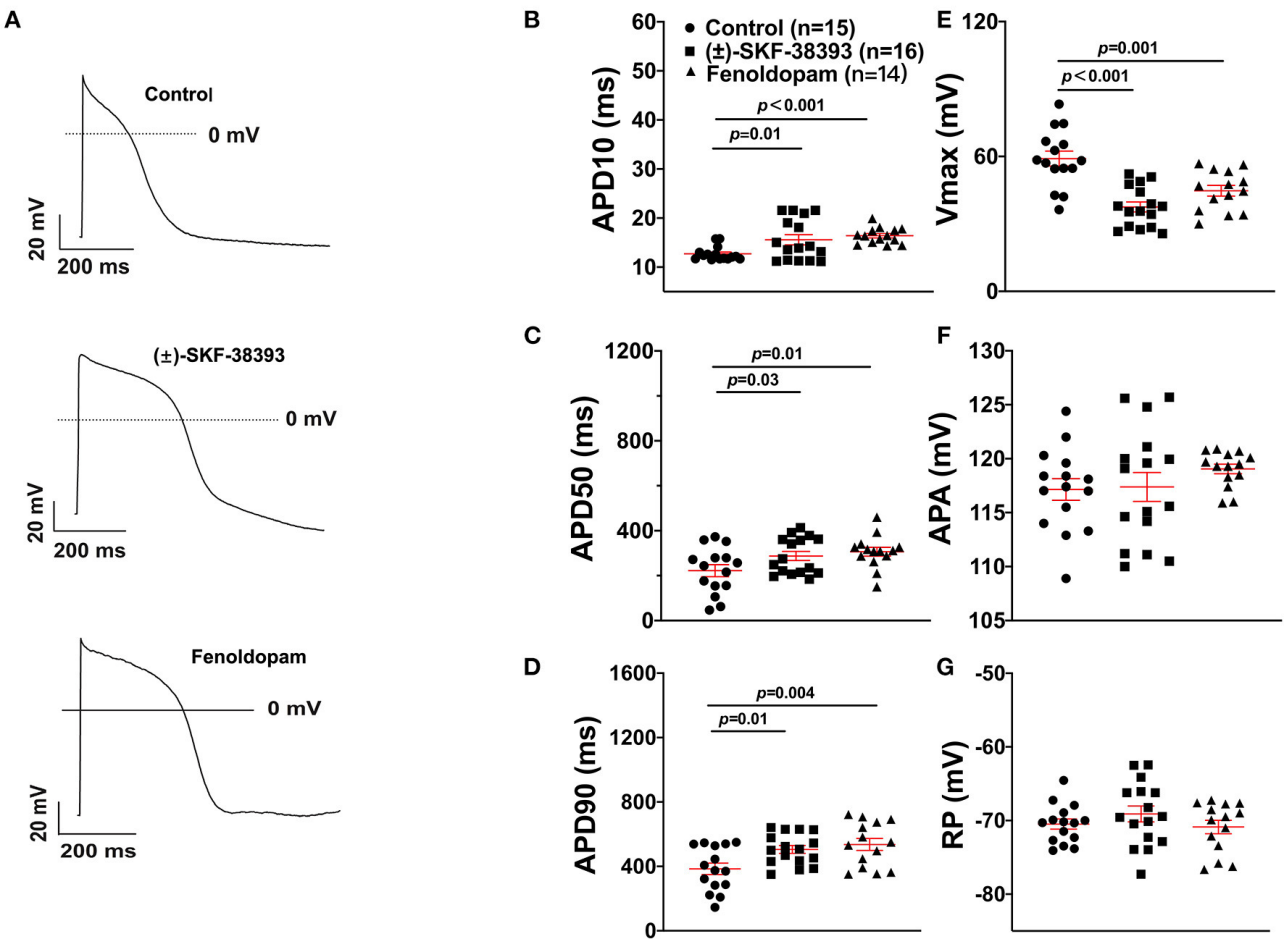
Dopamine D1/D5 receptor agonists mimicked epinephrine effects on the action potential (AP). hiPSC-CMs were challenged by either vehicle (Control) or 50 μM (±)-SKF 38393 or 5 μM fenoldopam for 1 h and action potentials (APs) were recorded. **(A)** Examples of AP traces in each group cells. **(B)** Mean values of APD10 in each group cells. **(C)** Mean values of APD50 in each group cells. **(D)** Mean values of APD90 in each group cells. **(E)** Mean values of AP Vmax in each group cells. **(F)** Mean values of AP amplitude (APA) in each group cells. **(G)** Mean values of resting potential (RP) in each group cells. “*n*” numbers in **(B)** are for **(B–G)**. The *p*-values vs. Control were determined by the analysis of one-way ANOVA with Holm-Sidak post-test.

To further confirm the role of dopamine D1/D5 receptor for AP-changes, (±)-SKF 38393 (50 μM) and fenoldopam (5 μM), the dopamine D1/D5 receptor specific agonists, were applied to hiPSC-CMs. Indeed, they mimicked Epi-effects (APD-prolongation and Vmax-suppression) (Figure 3). Taking all the data together, dopamine D1/D5 receptor signaling contributed to the Epi-effects on APs.

### ROS Mediated the Effects of the Dopamine D1/D5 Receptor Activation

**Figure 4:**
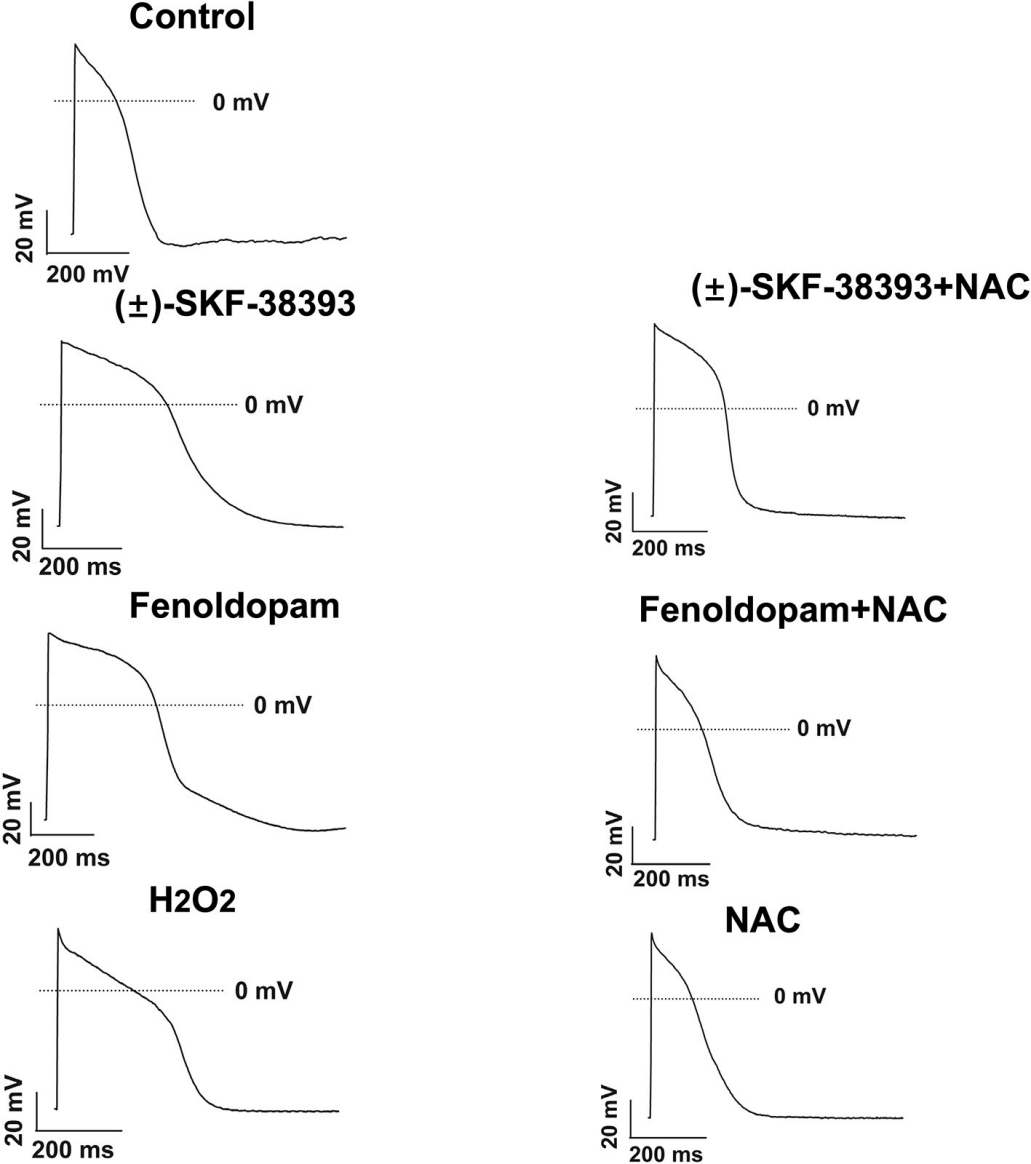
ROS mediated the effects of (±)-SKF 38393 or fenoldopam on action potentials. hiPSC-CMs were challenged by vehicle (Control) or 50 μM (±)-SKF 38393 and 5 μM fenoldopam or 100 μM H_2_O_2_ (H_2_O_2_) in the presence or absence of a ROS-blocker (1 mM NAC), or NAC alone for 1 h. APs were recorded at 1 Hz. Shown are examples of APs in each group cells. Statistical data were summarized in Table 2.

Our previous study detected that the activation of both α and β-adrenoceptors contributed to the ROS (reactive oxygen species) generation induced by the high concentration of isoprenaline and epinephrine, which led us to examine whether ROS also participates in the effects of dopamine D1/D5 receptor activation in catecholamine surge. First, we examined the effects of a ROS blocker *N*-acetylcysteine (NAC) on APs in presence of (±)-SKF 38393 or fenoldopam. Actually, NAC reduced and H_2_O_2_ (the main form of endogenous ROS) recapitulated the (±)-SKF 38393 and fenoldopam effects on APDs (Figure 4; Table 2), which are indicative of an involvement of ROS in effects of dopamine D1/D5 receptor activation. NAC alone displayed no effect on APs.

**Table 2:** Analysis of action potentials influenced by ROS in presence of dopamine receptor agonists.

**Group**	**Cell number**	**APD10**	**APD50**	**APD90**	**Vmax**	**APA**	**RP**
Control	16	12.9 ± 0.4	210.9 ± 27.4	369.2 ± 36.9	56.9 ± 3.8	117.6 ± 1.0	−70.4 ± 1.1
(±)-SKF-38393	18	16.2 ± 1.3^**^	333.3 ± 44^*^	566.1 ± 47.4^**^	39.1 ± 2.2^**^	118.1 ± 2.9	−71.6 ± 1.6
Fenoldopam	14	16.4 ± 0.4^*^	328.3 ± 29.8^*^	535.9 ± 36.9^**^	44.7 ± 2.4^**^	119.1 ± 0.4	−70.9 ± 0.9
H_2_O_2_	15	16.4 ± 0.5^*^	335.8 ± 16.7^*^	544.9 ± 35.8^**^	33.1 ± 1.2^**^	117.3 ± 1.5	−71.9 ± 0.8
(±)-SKF-38393+NAC	16	12.9 ± 0.3	220.7 ± 4.9	417.4 ± 5.2	47.4 ± 50.	117.8 ± 0.7	−69.5 ± 0.4
Fenoldopam + NAC	16	12.8 ± 0.26	233.6 ± 5.9	420.1 ± 7.6	47.6 ± 0.99	118.91 ± 0.5	−69.8 ± 0.5
NAC	16	12.8 ± 0.3	227.9 ± 5.5	433.7 ± 14.6	54.9 ± 1.31	118.5 ± 1.7	−68.3 ± 0.6

* *p < 0.05*,

** *p < 0.01. The p-values vs. control were determined by one-way ANOVA with Holm-Sidak post-test*.

**Figure 5:**
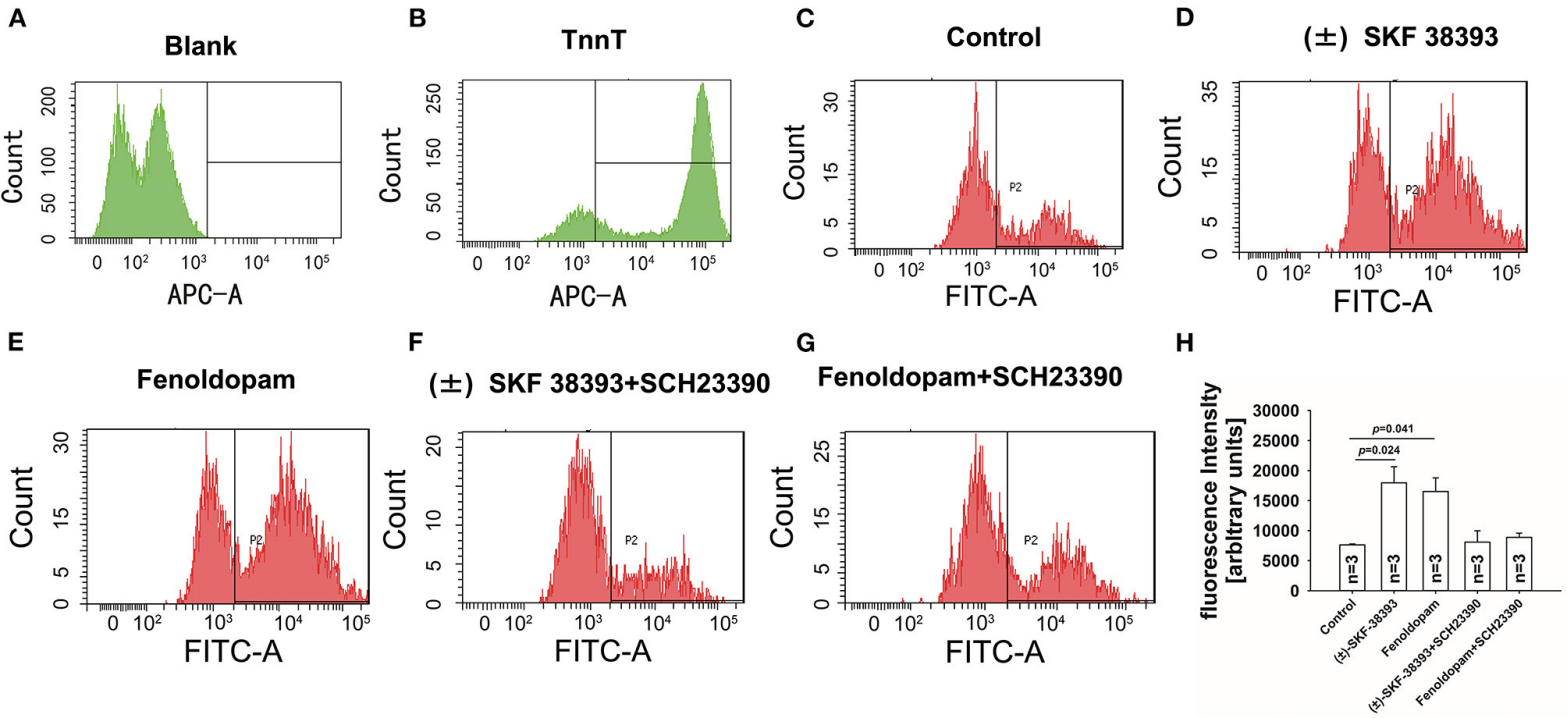
Dopamine D1/D5 receptor activation increased ROS-production. hiPSC-CMs were treated with or without antibody against troponin T (cTnT) and ROS fluorescence dye was applied to measure ROS production. Vehicle (Control) **(C)** or 50 μM (±)-SKF 38393 or 5 μM fenoldopam or (±)-SKF 38393 plus 10 μM SCH 23390 or fenoldopam plus 10 μM SCH 23390 **(D–G)** was applied to cells for 1 h before measurements. ROS-generation was analyzed by FACS. **(A,B)** A representative recoding indicating cardiac troponin T (cTnT) in hiPSC-CMs. **(D,E)** A representative recoding indicating enhancement of ROS generation by (±)-SKF 38393 or fenoldopam. **(F,G)** A representative recoding indicating that SCH 23390 prevented the effect of (±)-SKF 38393 or fenoldopam on ROS generation. **(H)** Averaged values of fluorescence intensity showing the ROS-levels in cells. “blank” means control measurements in cells that were not treated by the ROS fluorescence dye. “*n*” numbers in **(H)** are numbers of different independent experiments using cells from different differentiations. The *p*-values vs. Control were determined by the analysis of one-way ANOVA with Holm-Sidak post-test.

Next, the ROS level was measured by FACS in hiPSC-CMs in presence of (±)-SKF 38393 or fenoldopam. The ROS-level was increased in (±)-SKF 38393- or fenoldopam-treated cells (Figures 5C–E,H), suggesting that (±)-SKF 38393 or fenoldopam may affect APs through ROS. As expected, the dopamine D1/D5 receptor blocker reduced the (±)-SKF 38393 or fenoldopam-induced ROS generation in hiPSC-CMs (Figures 5F–H), which suggests that that ROS signaling may contribute to effects of dopamine D1/D5 receptor activation.

### NADPH Oxidases Were Involved in Effects of Dopamine D1/D5 Receptor Activation

**Figure 6:**
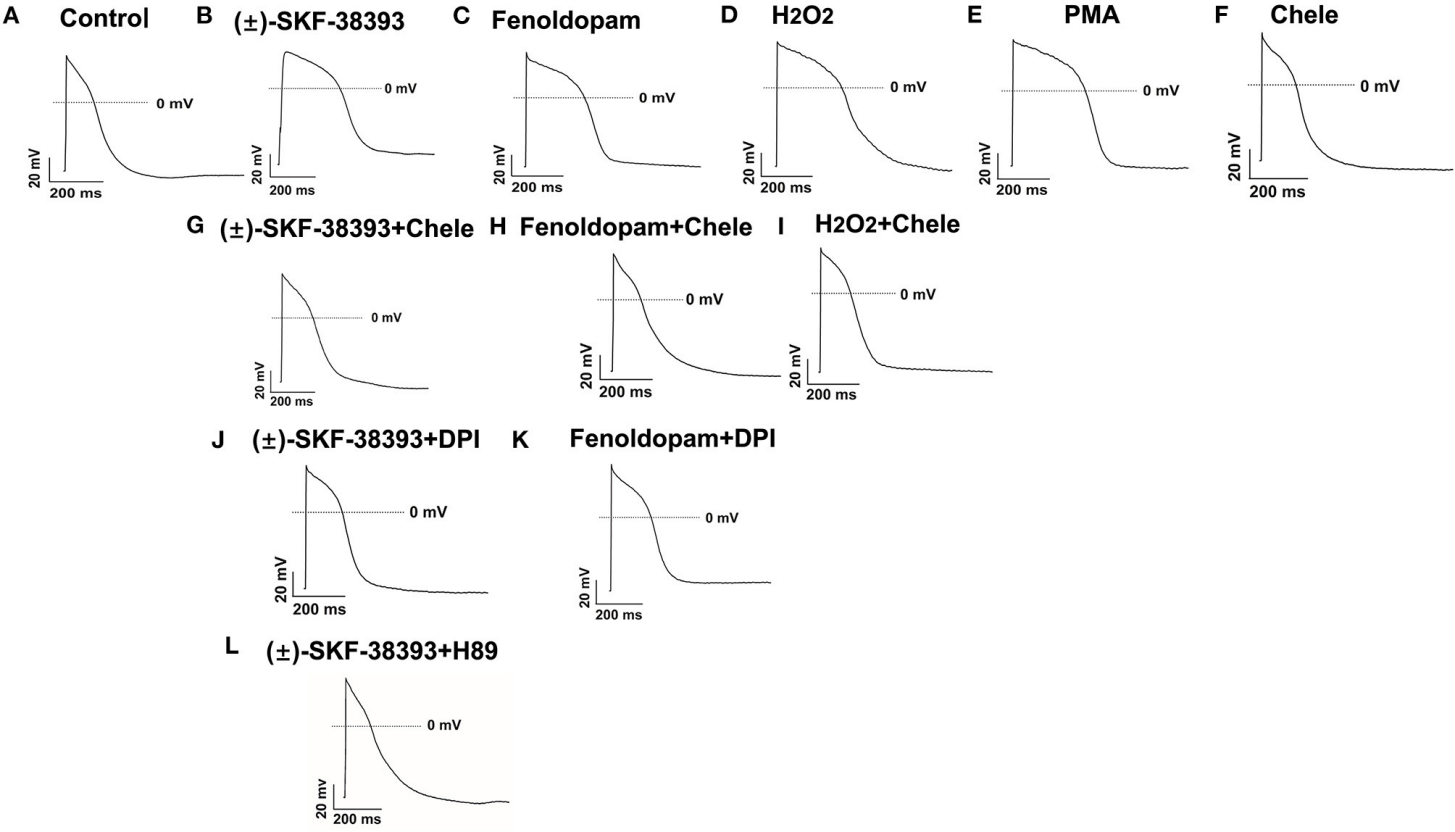
Protein kinases and NADPH oxidases participating in dopamine D1/D5 receptor and ROS signaling. hiPSC-CMs were challenged for 1 h by vehicle [Control, **(A)**] or 50 μM (±)-SKF 38393 **(B)** or 5 μM fenoldopam **(C)** or 100 μM H_2_O_2_ [H_2_O_2_, **(D)**] or 10 μM PKC activator PMA [PMA, **(E)**] or 10 μM Chele **(F)** or (±)-SKF 38393 plus 10 μM chelerythrine (Chele), an inhibitor of protein kinase C **(G)**, or fenoldopam plus Chele **(H)** or H_2_O_2_ plus Chele **(I)** or (±)-SKF38393 plus 10 μM DPI **(J)** or fenoldopam plus DPI **(K)** or (±)-SKF 38393 plus 1 μM H89, an inhibitor of protein kinase A **(L)**. Action potentials paced at 1 Hz were recorded. Statistical data are summarized in Table 3. **(A–L)** Examples of AP-traces in each group cells.

Considering that ROS generation is linked to numerus intracellular signaling, we intended to explore a signaling factor that may be responsible for the elevated ROS generation caused by activation of dopamine D1/D5 receptor. We found that diphenyleneiodonium (DPI), an inhibitor of NADPH oxidase, abolished the effects of (±)-SKF 38393 or fenoldopam on APs, suggesting that NADPH oxidases were involved in effects of dopamine D1/D5 receptor activation (Figures 6A–C,J,K; Table 3).

**Table 3:** Analysis of action potentials influenced by ROS, PKC, and PKA signaling in presence of dopamine receptor agonists.

**Group**	**Cell number**	**APD10**	**APD50**	**APD90**	**Vmax**	**APA**	**Baseline**
Control	16	12.9 ± 0.4	210.9 ± 27.4	369.2 ± 36.9	56.9 ± 3.8	117.6 ± 1.0	−70.4 ± 1.1
(±)-SKF-38393	18	16.2 ± 1.3^**^	333.3 ± 44^**^	566.1 ± 47.4^**^	39.1 ± 2.2^**^	118.1 ± 2.9	−71.6 ± 1.6
Fenoldopam	14	16.4 ± 0.4^**^	328.3 ± 29.8^**^	535.9 ± 36.9^**^	44.7 ± 2.4^**^	119.1 ± 0.4	−70.9 ± 0.9
H_2_O_2_	15	16.4 ± 0.5^**^	335.8 ± 16.7^**^	544.9 ± 35.8^**^	33.1 ± 1.2^**^	117.3 ± 1.5	−71.9 ± 0.8
PMA	11	15.8 ± 0.5^**^	303.7 ± 9.4^**^	533.0 ± 6.9^**^	42.6 ± 2.1^**^	117.8 ± 1.3	−68.4 ± 0.8
(±)-SKF-38393+H89	15	12.7 ± 0.3	170.5 ± 10.9	410.5 ± 24.7	60.9 ± 4.3	116.1 ± 0.9	−69.0 ± 0.6
(±)-SKF38393+Chele	18	13.2 ± 0.3	178.2 ± 24.7	386.3 ± 25.9	56.4 ± 2.2	117.4 ± 0.6	−69.5 ± 0.5
Fenoldopam+Chele	15	12.7 ± 0.4	221.3 ± 9.1	354.8 ± 30.9	59.6 ± 2.3	117.2 ± 1.1	−70.4 ± 0.7
H_2_O_2_+Chele	15	13.2 ± 0.3	211.6 ± 4.5	419.5 ± 9.2	52.6 ± 1.1	117.3 ± 0.7	−68.2 ± 0.4
(±)-SKF-38393+DPI	16	13.6 ± 0.3	218.9 ± 3.9	411.6 ± 9.4	44.1 ± 2.2	117.9 ± 0.6	−70.0 ± 0.8
Fenoldopam+DPI	13	12.6 ± 0.4	233.9 ± 5.8	429.5 ± 8.2	41.1 ± 1.5	118.9 ±±0.5	−69.0 ± 0.5
Chele	8	11.9 ± 0.6	214.6 ± 5.5	400.3 ± 6.5	54.6 ± 0.3	118.3 ± 0.3	−71.2 ± 0.4

** *p < 0.01. The p-values vs. Control were determined by one-way ANOVA with Holm-Sidak post-test*.

### Protein Kinase A (PKA) and C (PKC) Were Involved in the Effects of Dopamine D1/D5 Receptor Activation

It has already been demonstrated that stimulation of the D1-like receptors can activate the cyclic adenosine monophosphate (cAMP) and protein kinase A (PKA) signaling pathway (28, 29). But some studies also found that dopamine D1/D5 receptor can be coupled to Gq-PLC signaling and protein kinase C (PKC) is an important downstream signaling protein (30–32). We examined the possible roles of PKC and PKA for AP-changes. We found that a PKC-inhibitor (chelerythrine, 10μM) reduced the effect of dopamine D1/D5 receptor agonists on APs, similar to H89 (1 μM), a PKA-inhibitor (Figures 6A–C,G,H,L, Table 3). Using chelerythrine alone showed no effect on APs (Figure 6F). In addition, a PKC stimulator (PMA, 10 μM) displayed effects on APs similar to that of (±)-SKF 38393 (50 μM) or fenoldopam (5 μM) (Figures 6A–C,E; Table 3), implying that both PKA and PKC are required for the effect of dopamine D1/D5 receptor activation.

Given that ROS is a well-known downstream factor of PKA (33), we tried to clarify relationship between PKC and ROS in dopamine D1/D5 receptor activation. The hiPSC-CMs were treated with H_2_O_2_ with and without the PKC-inhibitor. Indeed, the PKC-inhibitor suppressed the effects of H_2_O_2_ (Figures 6D,I; Table 3), suggesting that PKC was a downstream signaling factor of ROS in the signal pathway.

### The Ionic Mechanisms of AP-Changes Caused by Dopamine D1/D5 Receptor Activation

To examine which ion channel dysfunctions may contribute to arrhythmogenesis mediated by dopamine receptors in the setting of TTC, we first assessed the ion channel expression profile in hiPSC-CMs challenged by high concentration (500 μM) epinephrine (Epi) plus dopamine D1/D5 receptor blocker SCH 23390 (Supplementary Figure 2). The expression level of CACNA1C (coding L-type calcium channel) genes was increased by Epi (Supplementary Figure 2A), but the expression level of SCN5A (coding Nav1.5 sodium channel) and KCNH2 (coding I_Kr_ channel) gene was reduced by Epi (Supplementary Figures 2B,C). The dopamine D1/D5 receptor blocker SCH 23390 (10 μM) attenuated the Epi-effect on gene expression level (Supplementary Figure 2), suggesting that the dopamine D1/D5 signaling may contribute to ion channel dysfunctions *via* changing ion channel expression levels in the setting of TTC.

**Figure 7:**
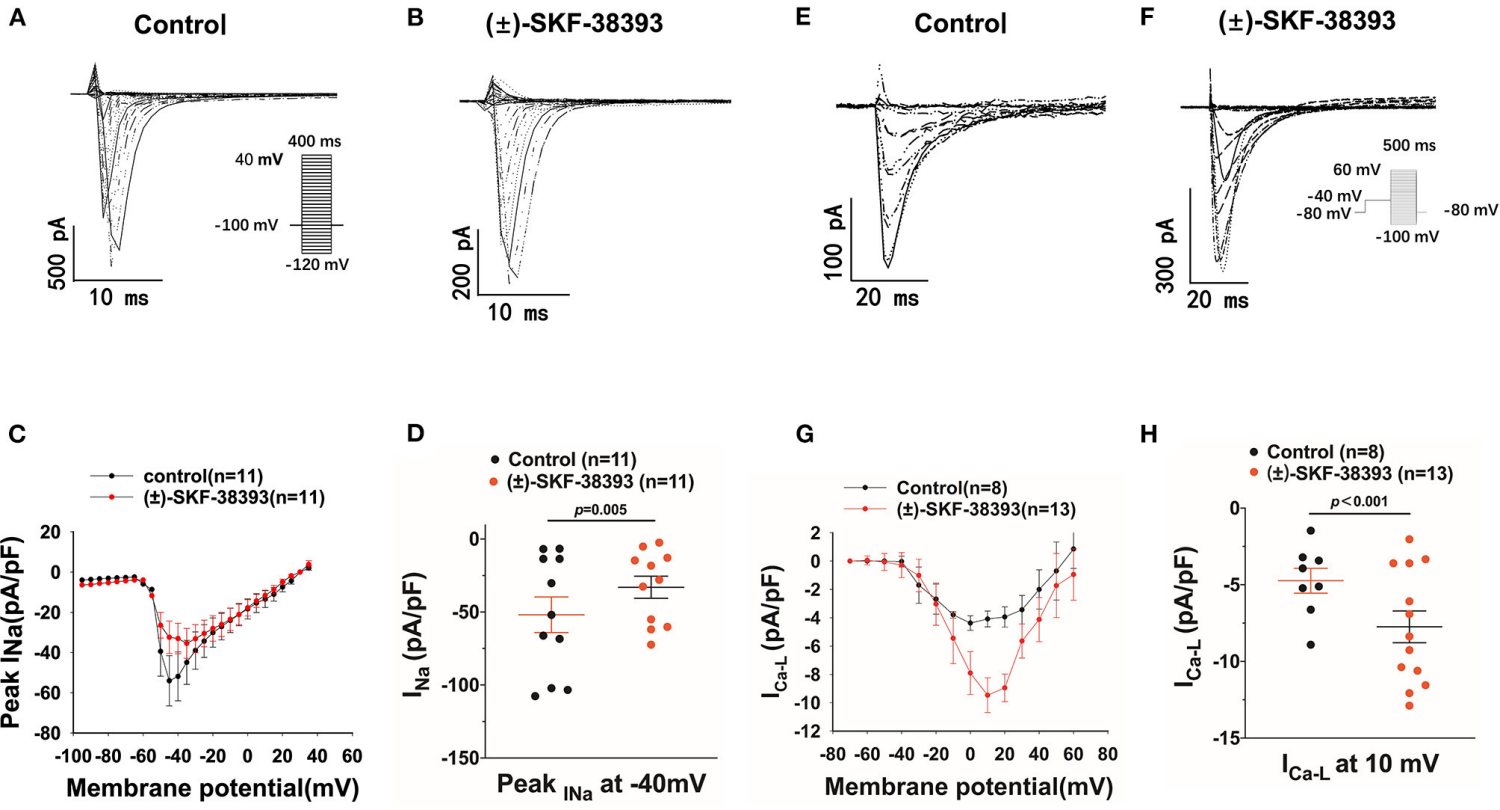
Effects of (±)-SKF 38393 or fenoldopam on ion channel currents. hiPSC-CMs were challenged for 1 h by vehicle (Control) or 50 μM (±)-SKF 38393 and 5 μM fenoldopam. Sodium channel current (I_Na_) was recorded with the patch clamp (voltage-clamp) protocol indicated in **(A)** (inset). The L-type calcium channel current (I_Ca−L_) was recorded with the voltage-clamp protocol indicated in **(F)** (inset). **(A,B)** Representative traces of I_Na_ in absence (Control) and presence of (±)-SKF38393. **(C)** I–V curves of peak I_Na_. **(D)** Mean values of peak I_Na_ at −40 mV. **(E,F)** Examples of traces of I_Ca−L_ in absence (Control) and presence of (±)-SKF38393. **(G)** I–V curves of I_Ca−L_. **(H)** Mean values of I_Ca−L_ at 10 mV. “*n*” numbers represent the number of analyzed cells. The *p*-values vs. Control were determined by *t*-test.

Furthermore, we evaluated the effects of (±)-SKF 38393 on ion channel currents. First, the effects of (±)-SKF 38393 on inward currents including I_Na_, I_CaL_ were examined in vehicle- (control) or (±)-SKF 38393-treated cells. (±)-SKF 38393 reduced the peak I_Na_ (Figures 7A–D) without influencing the voltage-dependent inactivation and activation and recovery of channels (Supplementary Figure 3).

(±)-SKF 38393 significantly increased the L-type calcium channel current (I_Ca−L_) (Figures 7E–H) without influencing the channel activation and inactivation of I_Ca−L_ (Supplementary Figures 4A–D). However, (±)-SKF 38393 strongly accelerated the time course of recovery of I_Ca−L_ (Supplementary Figures 4E,F).

Next, we observed that (±)-SKF 38393 decreased I_Kr_ (the rapidly activating delayed rectifier) and inhibited the channel activation by shifting the activation curve to a more positive potential (Supplementary Figures 5A–E).

In order to examine possible variations of experimental results among different cell lines, we repeated some pivotal experiments in hiPSC-CMs from other two healthy donors (H2 and H3). The results exhibited that in both H2 (Supplementary Figure 6) and H3 (Supplementary Figure 7) hiPSC-CMs, the dopamine D1/D5 receptor activators (±)-SKF 38393 and fenoldopam altered APs in the same manner as in the first healthy donor (H1) hiPSC-CMs. Either the positive (significant effects) and negative (no effects) result observed in H1-hiPSC-CMs was successfully recapitulated in H2- and H3-hiPSC-CMs, which means that the interindividual variation is not large under our experimental conditions.

## Discussion

Although there are clinical case reports demonstrating that dopamine injection can induce TTC or the plasma dopamine concentration of TTC patients increases, and experimental data showing that the use of high concentrations of dopamine successfully induced TTC in animal models, it is still unclear how dopamine receptor activation causes TTC in the human body. To our knowledge, this is the first study to explore the roles and mechanisms of dopamine D1/D5 receptor activation for APD/QT-prolongation and arrhythmias induced by toxic concentration of catecholamine using hiPSC-CMs.

It has been confirmed in animals that the stimulatory effect of epinephrine can be inhibited by the D1 antagonist SCH 23390 (34), suggesting that epinephrine can also activate dopamine receptors. Another study showed that norepinephrine and epinephrine both increased (35) SGTPgammaS binding at dopamine D4 receptors (35), suggesting an agonism of norepinephrine and epinephrine at the dopamine D4 receptor. Conversely, dopamine can also activate adrenoceptors (36, 37). These data may suggest an important cross-reactivity among the different monoamine neurotransmitter systems. To investigate the possible contributions of dopamine D1/D5 receptor signaling to catecholamine effects in the setting of TTC, we first used high concentration of epinephrine (Epi) to establish the TTC-model and examined effects of dopamine D1/D5 receptor blockers in the study. The D1/D5 antagonist SCH 23390 attenuated the Epi-induced arrhythmia in hiPSC-CMs. Moreover, both D1/D5 dopamine receptor agonist, (±)-SKF 38393 and fenoldopam, increased the arrhythmic events, indicative of the involvement of dopamine D1/D5 receptor signaling in occurrence of arrhythmias in the setting of TTC. The contribution of dopamine receptors in TTC can be understood in two aspects: (1) High concentration epinephrine can activate dopamine receptor through cross-activation; (2) In stress, catecholamine including dopamine release is largely increased and the stimulation of dopamine receptors by dopamine *per se* is enhanced, which enhances catecholamine effects.

Abnormal APs are usual substrates of arrhythmias, leading us to examine changes of APs caused by dopamine receptor activation. The dopamine receptor blocker SCH 23390 attenuated the effects of epinephrine and two specific agonists of dopamine D1/D5 receptor [(±)-SKF38393 and fenoldopam] mimicked the effect of Epi on APs. These data imply that dopamine D1/D5 receptor signaling can contribute to effects of epinephrine, promoting the changes of APs caused by high doses of catecholamine. The reduction of Vmax may cause conduction defect. The APD-prolongation may cause EADs. Both changes caused by D1/D5 receptor activation may contribute to occurrence of arrhythmic events, which is reported by TTC cases in up to 12% of patients (38, 39). Of note, epinephrine and dopamine agonists caused also DAD-like events and failed APs. The former suggests abnormal calcium-release or abnormal inward current, while the latter suggests abnormal automaticity in hiPSC-CMs challenged by toxic catecholamine.

The APD-prolongation induced by epinephrine is not consistent with previous reported data showing a shortening of APD by adrenergic stimulators (40). It is well-known that adrenergic stimulation increases heart rate and shortens QT interval and APD, which is a physiological frequency-adaptation. The heart rate acceleration results from activation of I_f_ channels by increased cAMP due to ß1-Gs activation. The QT/APD-shortening was shown to be caused by enhanced I_Ks_ (41). Of note, those effects result from adrenergic stimulation under physiological condition or at low level of catecholamine. Toxic effects of high level of catecholamine can be different from the physiological effects of catecholamine. Indeed, our study detected an opposite effect, i.e., an APD-prolongation in presence of high concentration of epinephrine. The reasons for the difference between physiological and toxic effects might be: (1) Receptor signaling switch. Low concentration catecholamine predominantly stimulates ß1-Gs-signaling, while high concentration catecholamine mainly stimulates ß2-Gi-siganling (42, 43); (2) Target switch. Low concentration catecholamine activates I_Ks_ (41), while high concentration catecholamine inhibits I_to_ (10) and I_Kr_ (this study); (3) Multiple effects. High concentration catecholamine stimulates more receptors and more signaling than low concentration catecholamine. Different signaling may interact each other and lead to either an enhancing or a counteracting effect. Therefore, the result in the current study can be understood as: high concentration epinephrine stimulates ß- (mainly ß2), α- and D1/D5-receptors. The ß1- (frequency enhancing, I_Ks_ enhancing and APD-shortening) effect was counteracted by ß2- or α- or even D1/D5-effects. The ß2- or α- or even D1/D5-effects on I_to_, I_Kr_, I_Na_, and I_Ca−L_, all facilitate APD-prolongation (10, 11).

Here, a question is whether the result difference between this and previous studies was caused by the immaturity of hiPSC-CMs. Our recent study showed that our hiPSC-CMs displayed frequency enhancement and APD-shortening when they were stimulated by a low (10 μM) concentration of isoprenaline (44), which are in agreement with previously reported data. This strongly supports that hiPSC-CMs have basic properties similar to human native cardiomyocytes and can provide helpful information in researches regarding cardiac physiology or pathophysiology.

Another question is what the importance and relationship of different receptor activation are in presence of high concentration catecholamine. Notably, our recent studies showed that blockade of ß-receptor or α-receptor could completely prevent the APD-prolongation by high concentration of isoprenaline and epinephrine, respectively (10, 11). High concentration of isoprenaline can probably activate multiple types of adrenoceptors. These data together with results of the current study demonstrated that the APD-prolongation induced by toxic catecholamine needs activation of multiple receptors because blockade of one type receptor can lead to loss of the effect. It implies that every involved receptor is important and activation of only one type of the receptor is not enough for causing APD-prolongation and arrhythmias.

Next, we investigated the ionic mechanism that causes AP changes. Inhibitory effects of Epi on SCN5A expression level and peak I_Na_ were reversed by dopamine D1/D5 receptor blocker (SCH 23390), Moreover, the (±)-SKF 38393 suppressed peak I_Na_ but did not change the channel gating kinetics. All data together indicated that the activation of dopamine D1/D5 receptors suppressed peak I_Na_ mainly *via* reducing the channel expression level. The inhibition of peak I_Na_ is the main reason for the observed reduction of Vmax of APs.

The L-type Ca channel current (I_Ca−L_) was increased by dopamine D1/D5 receptor activation *via* enhancing the channel activation and reducing the recovery time after inactivation. The mRAN level of L-type Ca channel (CACNA1C) was also elevated by Epi and inhibited by SCH 23390, which is consistent with the increase in I_Ca−L_ and suggests that dopamine D1/D5 receptor signaling can modulate both the channel activity and channel expression. The increase in I_Ca−L_ can explain the prolongation of APD. The enhanced I_Ca−L_ can increase the intracellular Ca^2+^ concentration, which may stimulate Ca^2+^ release from intracellular Ca^2+^-storage and in turn causes DADs or trigger activity.

I_Kr_ channels are important in cardiac repolarization, and their dysfunction can lead to prolongation of APD and QT interval, which can cause arrhythmias. It is well-known that some clinically used drugs have anti- or proarrhythmic effect (a side effect) due to their effect on I_Kr_. Therefore, examination of effect on I_Kr_ is widely performed in drug-screening for therapeutic or toxic effect. In hiPS-CMs, I_Kr_ blockers like sotalol and E-4031 prolonged APD (44), and gain-of-function of I_Kr_ due to a mutation in I_Kr_ gene shortened APD in hiPSC-CMs (24), indicating that I_Kr_ is an important repolarizing current in hiPSC-CMs. Hence, for exploring the reason for APD-prolongation by dopamine receptor activation, we examined I_Kr_. We observed that high concentration of Epi reduced KCNH2 (gene of I_Kr_) expression and dopamine receptor blocker attenuated Epi effect and that dopamine receptor agonist (±)-SKF 38393 suppressed significantly I_Kr_. The reduction of I_Kr_ can prolong APD and contribute to occurrence of arrhythmic events. Therefore, the suppression of I_Kr_ by dopamine D1/D5 receptor activation may contribute to APD/QT prolongation and arrhythmogenesis of TTC.

Besides I_Na_, I_Ca−L_, and I_Kr_ other currents may also be affected by high level catecholamine and dopamine receptor signaling. Our recent study showed that hiPSC-CMs express almost all the ion channels reported in human native cardiomyocytes (44). I_Na_, I_Ca−L_, I_Ca−T_, I_f_, I_NCX_, I_K1_, I_to_, I_Kr_, I_Ks_ I_KATP_, I_K−pH_, I_SK1−3_, and I_SK4_, I_KACh_ (acetylcholine activated K^+^ current) and I_KNa_ (Na^+^-activated K^+^ current), I_K−pH_ (pH-sensitive K^+^ current), I_Cl−vol_ (volume-regulated Cl^−^ current) and I_Cl−Ca_ (calcium-activated Cl^−^ current), and TRPV channel current could be detected in hiPSC-CMs. We did not examine other currents because the enhanced I_Ca−L_ and reduced I_Kr_ can explain the APD-prolongation. It is possible that some other currents could be also affected and contribute to the APD-prolongation in presence of high concentration catecholamine.

There is a remarkable relationship between dopamine D1/D5 receptor and oxidative stress in abnormal dopamine signaling in animal models (45, 46). To further investigate the signaling factors linked to dopamine D1/D5 receptor activation, we first assessed the ROS generation, which has been shown to be linked to dopamine D1/D5 receptor (32). After treating the hiPSC-CMs with (±)-SKF 38393 or fenoldopam, the ROS production was increased obviously. In addition, SCH 23390 abolished the increase of ROS production. To further confirm the relationship between ROS and dopamine receptors activation, we next examined the effect of ROS blocker NAC on APs in the presence of (±)-SKF 38393 and fenoldopam. The NAC attenuated and H_2_O_2_ (endogenous ROS) mimicked the effects of (±)-SKF 38393 and fenoldopam on APs. These data indicate an important role of ROS for AP-changes linked to dopamine D1/D5 receptor activation.

However, the increase in ROS level is related to many factors, and it is currently known that NADPH oxidases plays an important role in the process of ROS production. Therefore, we next analyzed the effect of DPI (a NADPH oxidase blocker) on the activation of dopamine D1/D5 receptor. The DPI attenuated the effects of (±)-SKF 38393 and fenoldopam, which means that NADPH oxidase is involved in the signal transduction of AP changes caused by the activation of dopamine D1/D5 receptors.

A large number of studies have shown that activation of G protein-linked pathways by dopamine can inhibit Na-K-ATPase activity involving protein kinase C (PKC) and ROS activation will cause PKC pathway activation (47, 48). So, we next examined the PKC effects in dopamine D1/D5 receptor activation. The PKC blocker and activator were used. The PKC-blocker (chelerythrine) reduced the effects of both (±)-SKF 38393 and fenoldopam and the PKC activator (PMA) recapitulated their effects on APs. These data confirmed an important role of PKC in dopamine D1/D5 receptor mediated effects.

Since we observed that PKA, PKC and ROS all were involved in the effect of D1/D5 receptor activation, we tried to unveil the signal transduction process. PKA is a known upstream regulator of ROS (33). Therefore, the question is whether PKC is a downstream or upstream factor of ROS when D1/D5 receptors are activated. We found that the PKC blocker abolished the ROS effect, indicating that PKC is a downstream factor of ROS. Considering that NADPH oxidases were also involved in effects of dopamine D1/D5 receptor activation since DPI prevented the effects of (±)-SKF 38393 and fenoldopam, we may interpret the signaling in dopamine D1/D5 receptor activation by high concentration of catecholamine as: dopamine D1/D5 receptor-Gs-PKA-NADPH-ROS-PKC. However, how PKA causes NADPH oxidase activation is unknown and further studies are required to answer the question.

## Conclusion

The study showed that excessive catecholamines can cause abnormal ion channel function and arrhythmias *via* activating dopamine D1/D5 receptor signaling. Activation of dopamine D1D5 receptors is related to PKA, ROS, and PKC related signals. Dopamine D1/D5 receptors may contribute to the occurrence of arrhythmia in patients with TTC and may be possible therapeutic targets for treating the disease.

## Study Limitations

In this study, we only studied the role of D1/D5 receptor, and the role of D2 receptor family was not investigated in this study. Possible contributions of D2 family to toxic effects of catecholamine cannot be excluded. In addition, there are many different PKC isozymes for protein kinase C (PKC). This study did not clarify the subtypes of PKC responsible for the observed effects mediated by D1/D5 receptor signaling.

Besides, this study used hiPSC-CMs only from healthy people. Whether hiPSC-CMs from TTC patients show different results is still unknown. Moreover, differences exist between hiPSC-CMs and adult cardiomyocytes, which should also be considered when interpreting the data from hiPSC-CMs. The hiPSC-CMs have automaticity and a relatively high percentage of control cells exhibited the arrhythmic events. This might reflect a limitation of hiPSC-CMs comparing with adult human cardiomyocytes.

Another limitation of the study is that the APs and currents were measured at room temperature. The possibility that at higher temperature different effects of catecholamine may appear cannot be excluded.

## Data Availability Statement

The original contributions presented in the study are included in the article/Supplementary Material, further inquiries can be directed to the corresponding author/s.

## Ethics Statement

The skin biopsies were obtained from three healthy donors after written informed consent was accumulated. The Ethics Committee of the Medical Faculty Mannheim, University of Heidelberg (approval number: 2018-565N-MA) and the Ethics Committee of the University Medical Center Göttingen (approval number: 10/9/15) have approved the study. The study was performed following the approved guidelines and carried out according to the Helsinki Declaration of 1975 (https://www.wma.net/what-we-do/medical-ethics/declaration-of-helsinki/), revised in 2013. The patients/participants provided their written informed consent to participate in this study.

## Author Contributions

MH, YL, ZY, LC, HL, GY, and KB contributed to experiments, data collection, and data analysis. SL contributed to data analysis. SL, IE-B, LC, MB, XZ, and IA contributed to the conception or design of the work, interpretation of data, and writing the paper. All authors have read and approved the manuscript.

## Funding

This study was supported by the DZHK (German Center for Cardiovascular Research), the BMBF (German Ministry of Education and Research) (Nos. 81Z0500204 and 81X2500208), and the Hector Foundation (MED 1814).

## Conflict of Interest

The authors declare that the research was conducted in the absence of any commercial or financial relationships that could be construed as a potential conflict of interest.

## Publisher's Note

All claims expressed in this article are solely those of the authors and do not necessarily represent those of their affiliated organizations, or those of the publisher, the editors and the reviewers. Any product that may be evaluated in this article, or claim that may be made by its manufacturer, is not guaranteed or endorsed by the publisher.

## Abbreviations

TTC: Takotsubo cardiomyopathy
hiPSC-CMs: Human-induced pluripotent stem cell-derived cardiomyocytes
Epi: Epinephrine
DCFH-DA, 2′: 7′-Dichlorofluorescin diacetate
Chele: chelerythrine
I_Ca−L_: L-type (I_Ca−L_) calcium channel current
I_Kr_: rapidly activating delayed rectifier
EAD: early afterdepolarization
DAD: delayed afterdepolarization
APs: action potentials
APD10: action potential duration at 10% repolarization
APD50: action potential duration at 50% repolarization
APD90: action potential duration at 90% repolarization
Vmax: maximal depolarization velocity
RP: resting potential
APA: amplitude of action potential
ROS: reactive oxygen species
PKA: protein kinase A
PKC: protein kinase C
cAMP: cyclic adenosine monophosphate.
