# Supplementary material for: Dopamine D1/D5 Receptor Signaling Is Involved in Arrhythmogenesis in the Setting of Takotsubo Cardiomyopathy
Source: Front Cardiovasc Med. 2022 Feb 4;8:777463. doi: 10.3389/fcvm.2021.777463 (PMC8855058; doi:10.3389/fcvm.2021.777463)
Supplement: Supplementary file 1 [file Data_Sheet_1.docx]

**Supplementary information**

**Dopamine D1/D5 receptor signaling is involved in arrhythmogenesis in the setting of Takotsubo Cardiomyopathy**

**Running title:** **Dopamine receptor  in Takotsubo Syndrome**

Mengying Huang^a^, Zhen Yang^a^, Yingrui Li^a^, Huan Lan^b^, Lukas Cyganek^c,d^, Goekhan Yuecel^a,c^, Siegfried Lang^a,c^, Karen Bieback^e^, Ibrahim El-Battrawy^a,c^, Xiaobo Zhou^a,b,c*^, Martin Borggrefe^a,c#^ and Ibrahim Akin^a,c#^

^a^First Department of Medicine, University Medical Centre Mannheim (UMM), University of Heidelberg, Mannheim, Germany

^b^Key Laboratory of Medical Electrophysiology of Ministry of Education and Medical Electrophysiological Key Laboratory of Sichuan Province, Institute of Cardiovascular Research, Southwest Medical University, Luzhou, Sichuan, China

^c^DZHK (German Center for Cardiovascular Research), Partner Sites, Heidelberg-Mannheim and Göttingen, Germany

^d^Stem Cell Unit, Clinic for Cardiology and Pneumology, University Medical Center Göttingen, Göttingen, Germany

^e^Institute of Transfusion Medicine and Immunology, University Medical Centre Mannheim (UMM), University of Heidelberg, Mannheim, Germany

*Corresponding author

#Drs Akin and Borggrefe share senior authorship

**Address for correspondence**:

Xiaobo Zhou, First Department of Medicine, University Medical Centre Mannheim, Theodor-Kutzer-Ufer 1-3, 68167 Mannheim, Germany.

Email: Xiaobo.zhou@medma.uni-heidelberg.de

**Methods**

**Characterization and selection of human induced pluripotent stem cell-derived cardiomyocytes (hiPSC-CMs)**

The hiPSC-CMs from day 40 to day 60 were used for the study. Before application of hiPSC-CMs, characterizations of hiPSC-CMs were examined by different analyses. First, cardiac marker genes including MLY2, TNNT2, NKX2.5, ACTN2, and MYH6 were analyzed by qPCR to check the gene expression. Then, α-actinin and cardiac troponin (cTNT, or TnnT) protein expression was examined by immunostaining. Both analyses showed expression the cardiac markers in hiPSC-CMs. Further, functional studies including action potential and sodium channel (SCN5A) current were measured because iPS cells cannot generate action potential (APs) and they express no SCN5A channels. FACS analysis showed that more than 80% of cells after differentiation are cTNT positive. Patch clamp measurement showed that around 75% spontaneous beating cells showed ventricle-like, 20% cells showed atrial-like and 5% cell showed node-like APs. For AP-analyses, only cells with ventricle-like APs were included. For current measurements, APs were not recorded in the same cell. To reduce the possible bias in experiments, 1) cells of both the control and experimental groups were taken from the same cell culture flask, 2) cells were randomly grouped for control and treatments and 3) every group of cells were measured under same conditions.

## Fluorescence-activated cell sorting (FACS)

In FACS analysis, flow cytometry and fluorescent probes were used. To measure ROS level, 2′,7′-Dichlorofluorescin diacetate (DCFH-DA, sigma) was used. DCFH-DA is a non-polar fluorescence probe that can penetrate cell membranes, where it is converted into DCFH (excitation wavelength, 505 nm; emission wavelength, 526 nm) and then detected by flow cytometry. Fluorescein isothiocyanate (FITC) will absorb light from 400-530 nm but most efficiently 490 nm wavelength.

To measure TnnT, an anti-TnnT (APC anti- Rabbit Alexa 633) antibody was used. Allophycocyanin (APC) is a 105 kDa protein, which is maximally excited at 652 nm (with a secondary maximum at 625 nm) and emits at 658 nm.

The measurement was performed according to the protocol in following steps:

1. The cardiomyocytes were detached with 0.05% Trypsin-EDTA (Life Technologies) for 2- 4 min at 37 °C.
2. Thereafter, RPMI medium containing 10% FBS was added.
3. Cells were centrifuged at 250 × g for 4 min at room temperature.
4. Next, the supernatant was discarded and the cells were resuspended in basic culture medium.
5. The cells were added into the 15 ml tubes with 1 × 10^6^ cells/tube and incubated with 10 μM DCFH-DA (D6883; sigma) or anti- Rabbit Alexa 633(A21070; life technologies) at 37°C for 30 minutes in the dark. The cells were washed 3 times with PBS.
6. Acquire samples on a flow cytometer using a stop condition of 10,000 events on the gate of interest and ensure that FSC, SSC, ROS (FITC DCFH-DA) or TnnT (APC anti- Rabbit Alexa 633) channels are selected for the parameters to be analyzed.
7. Open the BD FACS Diva software (Version 8.0.1) and run the “unstained, untreated” sample. Then, open a dot plot for FSC (on the x-axis) vs SSC (on the y-axis) and draw a gate around the cells of interest, excluding dead cells and debris which are much smaller events than the main cell population and appear on the lower left of the plot.
8. Using this gate to select the cells in the gate and open the plot with ROS (FITC DCFH-DA, FITC-A) or TnnT (APC anti- Rabbit Alexa 633, APC-A) on the x-axis.

**Supplementary tables:**

**Supplementary table 1**

**Table S1. Primer sequences for real-time polymerase chain reaction (PCR).**

**Primer Company No**

Hs_CACNA1C_1_SG QuantiTect Primer Assay Qiagen QT000580

Hs_SCN5A_1_SG QuantiTect Primer Assay Qiagen QT00091812

Hs_KCNH2_2_SG QuantiTect Primer Assay Qiagen QT01003254

Hs_GAPDH_1_SG QuantiTect Primer Assay Qiagen QT00079247

Hs_DRD1_1_SG QuantiTect Primer Assay Qiagen QT00200025

Hs_DRD2_1_SG QuantiTect Primer Assay Qiagen QT00012558

Hs_DRD3_1_SG QuantiTect Primer Assay Qiagen QT00020307

Hs_DRD4_1_SG QuantiTect Primer Assay Qiagen QT00204316

Hs_DRD5_1_SG QuantiTect Primer Assay Qiagen QT0021765

**Supplementary table 2**

**Table S2. Antibody for Immunofluorescence (IF) staining.**

**Antibody Company No**

Alexa Fluor® 647 Mouse Anti-Cardiac- BD Biosciences 565744

Troponin T

Anti-Dopamine Receptor D1 antibody Abcam ab40653

Goat anti-Mouse IgG (H+L) Highly Cross- Thermofisher A32723

Adsorbed. Secondary Antibody, Alexa Fluor Plus 488

Anti-Cardiac Troponin T antibody Abcam ab8295

Goat anti-Rabbit IgG (H+L) Cross-Adsorbed- Thermofisher A21070

Secondary Antibody, Alexa Fluor 633

**Supplementary table 3**

**Table S3. Reagents and substances.**

| **Kits and substances** | **Company No** |
| --- | --- |
| RNeasy Mini Kit (250) | Qiagen 74106 |
| High-Capacity cDNA Reverse Transcription Kit | Thermo Fisher 4368814 |
| SibirRoxHot Master Mix, ROX 0.1μM | Bioron 119505 |
| VECTASHIELD with DAPI 10 ml | Biozol H-1200 |
| (±)-SKF-38393 hydrochloride | Sigma D047 |
| N-Acetyl-L-cysteine | Sigma A0737 |
| 2′,7′-Dichlorofluorescin diacetate | Sigma D6883 |
| Fenoldopam | Sigma SML0198 |
| R (+)-SCH-23390 hydrochloride | Sigma D054 |
| H_2_O_2_ (30%) | Merck 7722-84-1 |
| (±)-Epinephrine hydrochloride | Sigma E4642 |
| Chelerythrine chloride | Sigma 3895-92-9 |
| Phorbol 12-myristate 13-acetate | Sigma 16561-29-8 |
| Diphenyleneiodonium chloride | Sigma D2926 |

**Figure legends**

**Figure S1. Expression of dopamine receptors in hiPSC-CMs.**

(A) Averaged values of expression level (normalized to GAPDH) of dopamine receptors (DRD1, DRD2, DRD3, DRD4 and DRD5) assessed by qPCR analysis. (B) Immunostaining of hiPSC-CMs with antibodies against a cardiac marker cTnT (TnnT, green) and dopamine 1 receptor (DRD1, red), which is a representative member of D1/D5 receptor family. The nucleus was stained by DAPI (blue). The “n” numbers represent numbers of experiments from independent differentiations of cells.

**Figure S2. The dopamine D1/D5 receptor activation contributes to effects of epinephrine on ion channel expression.** HiPSC-CMs were treated for 1 h with either vehicle (Control) or 500 µM epinephrine (Epi) or epinephrine plus 10 µM SCH 23390 (dopamine receptor blocker). qPCR analysis was used to measure the expression levels of ion channels. The expression levels in presence of Epi or Epi+SCH 23390 were normalized to that in absence of Epi (A-C). (A) Mean values of L-type calcium channel (CACNA1C) expression. (B) Mean values of sodium channel (SCN5A) expression. (C) Mean values of rapidly activating delayed rectifier K (KCNH2, I_Kr_) expression. “n” numbers represent numbers of experiments from different independent differentiations of cells. The p values were determined versus Control according to the analysis of one-way ANOVA with Holm-Sidak post-test.

**Figure S3. Peak sodium channel gating kinetics in presence of SKF 38393.** HiPSC-CMs were treated for 1 h with vehicle (Control) or 50 µM (±)-SKF 38393. Peak I_Na_ was measured and gating kinetics including activation, inactivation and recovery from inactivation of peak I_Na_ were analyzed. (A) Protocol for recording I_Na_ at different potentials for analyzing activation curves. (B) Protocol for analyzing inactivation of I_Na_. (C) Protocol for analyzing recovery from inactivation of I_Na_, (D) Activation curves of I_Na_ from each group. (E) Mean values of potentials at 50% activation (V0.5). (F) Inactivation curves of I_Na_ from each group. (G) Mean values of potentials at 50% inactivation (V0.5). (H) Time course curves of I_Na_ recovery from inactivation. (I) Mean values of time constants (Tau) of recovery from inactivation. “n” numbers represent the number of cells. The p values were determined versus Control according to the analysis of t-test.

**Figure S4. L-type calcium channel kinetics was changed by (±)-SKF 38393.** HiPSC-CMs were treated for 1 h with vehicle (Control) or 50 µM (±)-SKF 38393. The L-type calcium channel current (I_Ca-L_) was measured and gating kinetics including activation, inactivation and recovery from inactivation were analyzed. (A) Protocol for recording I_Ca-L_ at different potentials for analyzing activation curves. (B) Protocol for analyzing inactivation of I_Ca-L_. (C) Protocol for analyzing recovery from inactivation of I_Ca-L_, (D) The activation curves of I_Ca-L_ in cells from each group. (E) The half-maximum activation potential (V0.5) of I_Ca-L_ in cells from each group. (F) The inactivation curves of I_Ca-L_ in cells from each group. (G) The half-maximum inactivation potential (V0.5) of I_Ca-L_ in cells from each group. (H) The curves of recovery from inactivation of I_Ca-L_ in cells from each group. (I) The time constants (tau) of recovery from inactivation of I_Ca-L_ in cells from each group. “n” numbers represent the number of cells. The p values were determined versus Control according to the analysis of t-test.

**Figure S5. Rapidly activating delayed rectifier currents (I_Kr_) was reduced by (±)-SKF 38393.** HiPSC-CMs were treated for 1 h with vehicle (Control) or 50 µM (±)-SKF 38393. I_Kr_ was recorded by using Cs^+^ as the charge carrier. The current was recorded with the voltage-clamp protocol indicated in A (inset). (A) Representative current traces of I_Kr_ in hiPSC-CMs treated with vehicle (Control) or (±)-SKF 38393. (B) I-V curves of rapidly activating delayed rectifier currents (I_Kr_). (C) Mean values of I_Kr_ at -30 mV. (D) The activation curves of I_Kr_. (E) The half-maximum activation potential (V0.5) of I_Kr_. “n” numbers represent the number of cells.

**Figure S6. hiPSC-CMs from the second donor mimicked results obtained in hiPSC-CMs from the first donor**. A second healthy donor (H2) was recruited and hiPSC-CMs were generated (H2-hiPSC-CMs). The cells were treated for 1 h with either vehicle (Control) or 50 µM (±)-SKF 38393 or 5 µM fenoldopam. (A) Representative AP-traces in a cell from each group. (B) Averaged values of APD10. (C) Averaged values of APD50. (D) Averaged values of APD90. (E) Averaged values of Vmax. (F) Averaged values of APA. (G) Averaged values of resting potential (RP). “n” numbers given in B represent the cell numbers for B-G. The p values were determined versus Control according to the analysis of one-way ANOVA with Holm-Sidak post-test.

**Figure S7. hiPSC-CMs from the third donor mimicked results obtained in hiPSC-CMs from the first donor.** A third healthy donor (H3) was recruited and hiPSC-CMs were generated (H3-hiPSC-CMs). The cells were treated for 1 h with either vehicle (Control) or 50 µM (±)-SKF 38393 or 5 µM fenoldopam. (A) Representative AP-traces in a cell from each group. (B) Averaged values of APD10. (C) Averaged values of APD50. (D) Averaged values of APD90. (E) Averaged values of Vmax. (F) Averaged values of APA. (G) Averaged values of resting potential (RP). “n” numbers given in B represent the cell numbers for B-G. The p values were determined versus Control according to the analysis of one-way ANOVA with Holm-Sidak post-test.

**Figure S1**


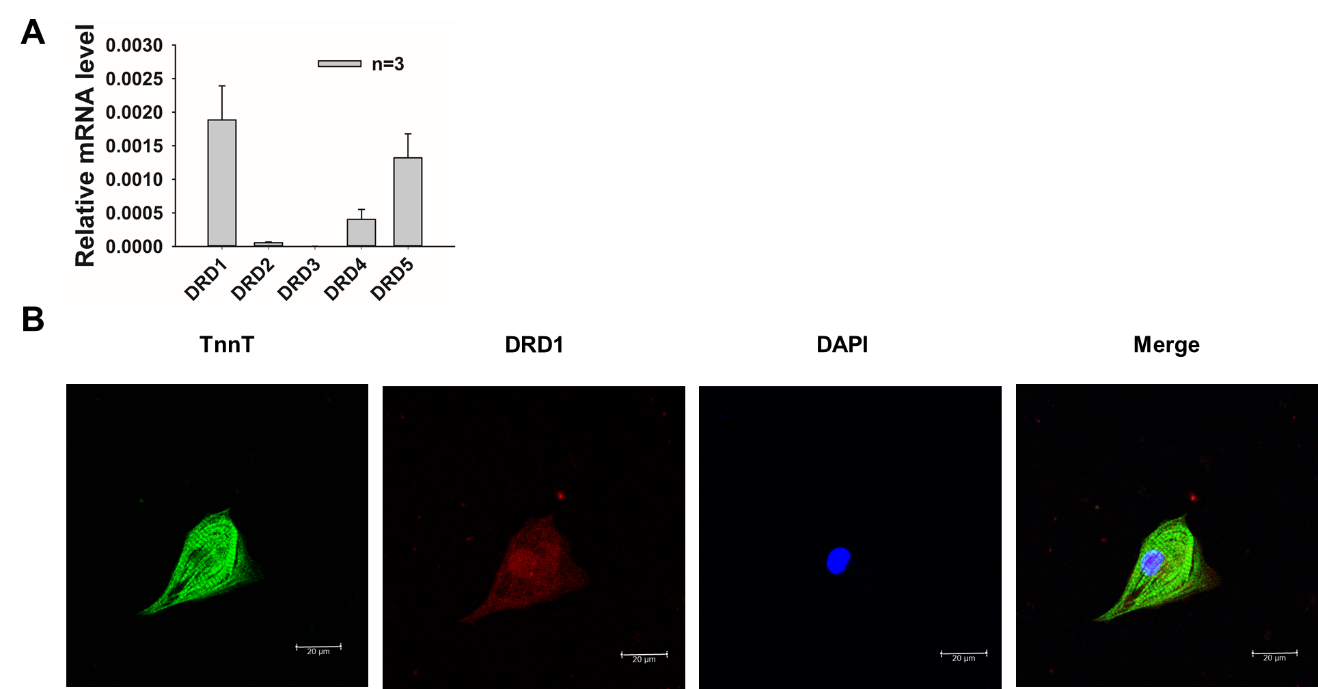


**Figure S2**


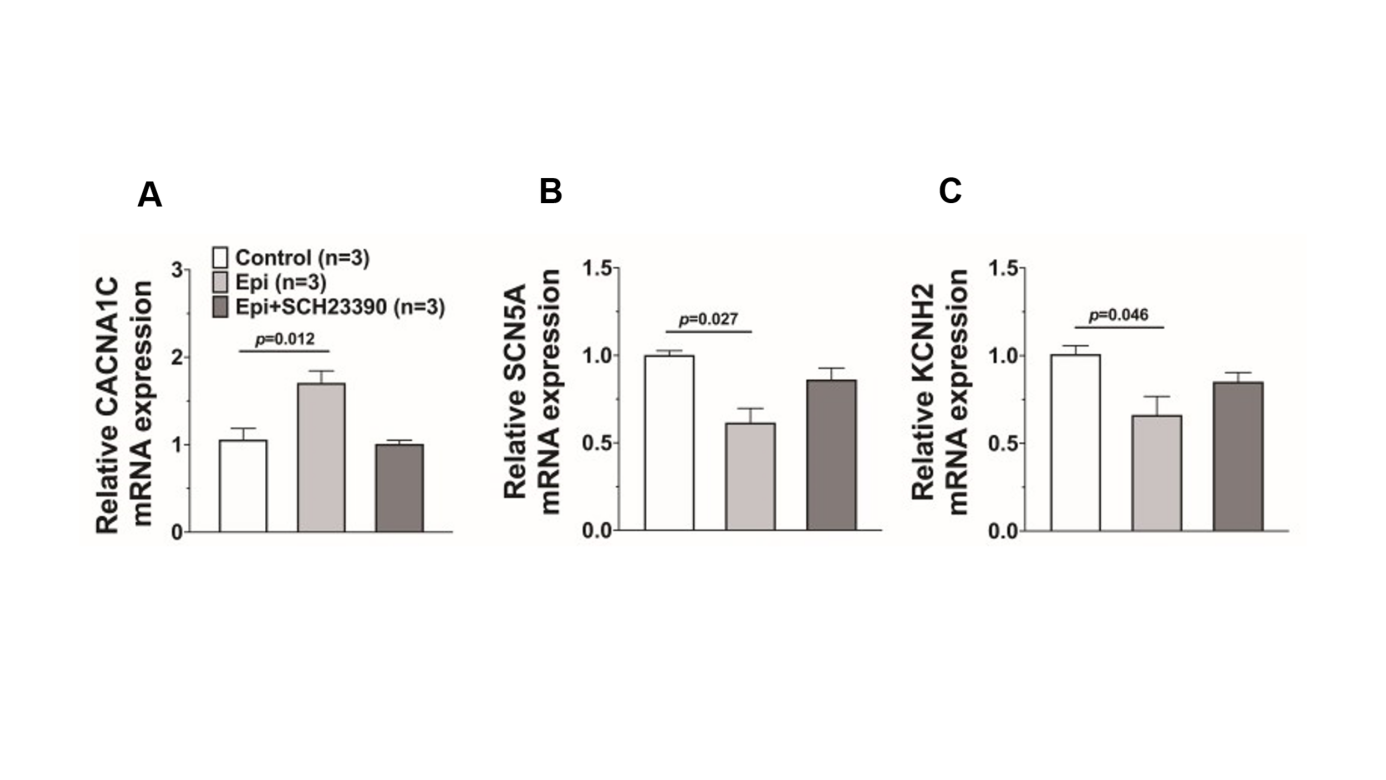


**Figure S3**


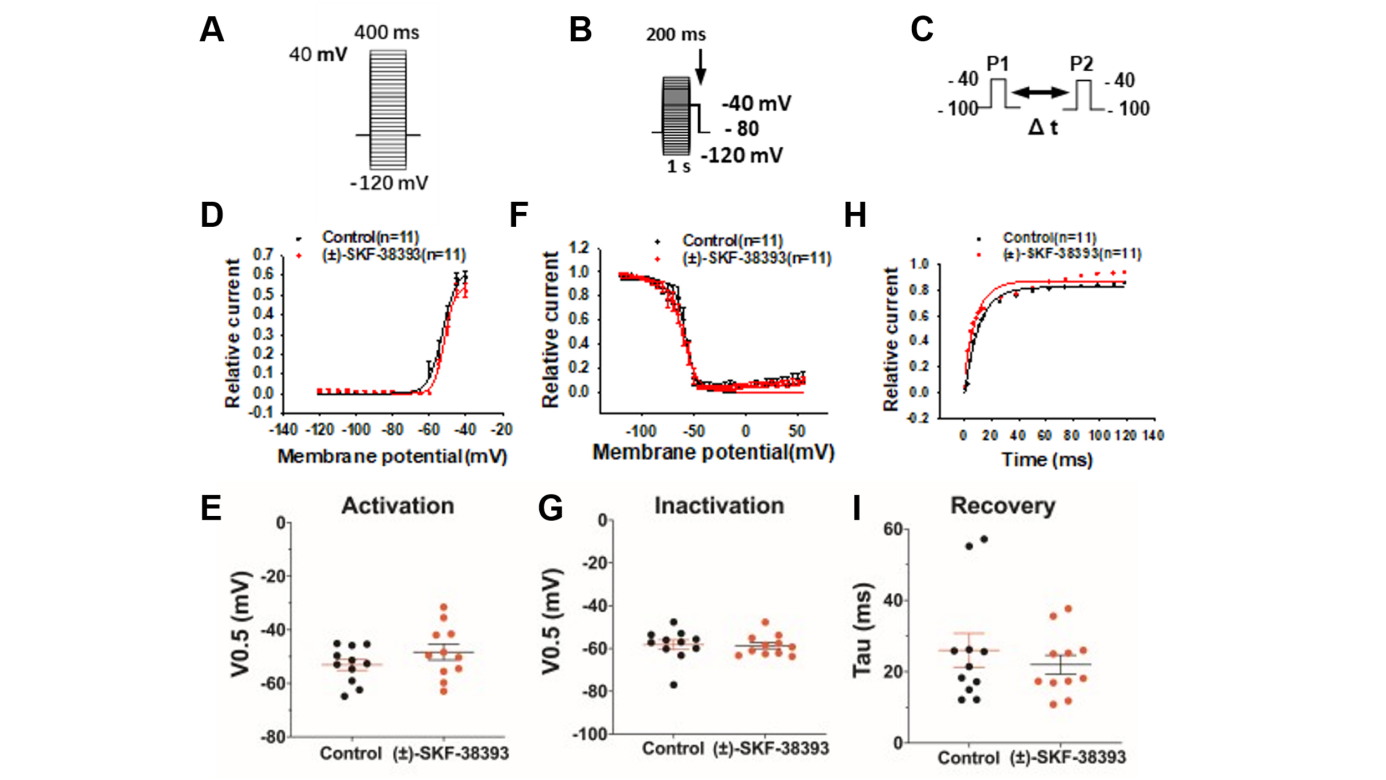


**Figure S4**


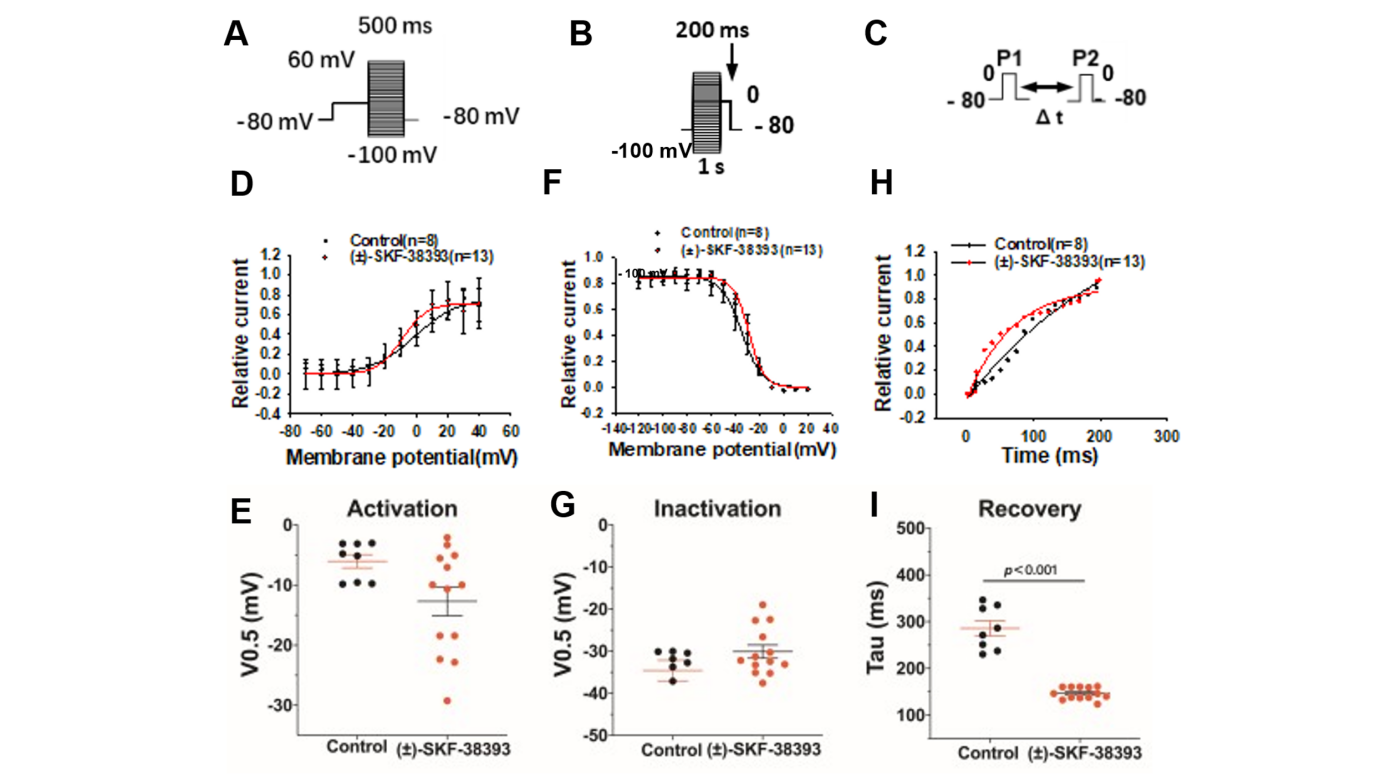


**Figure S5**


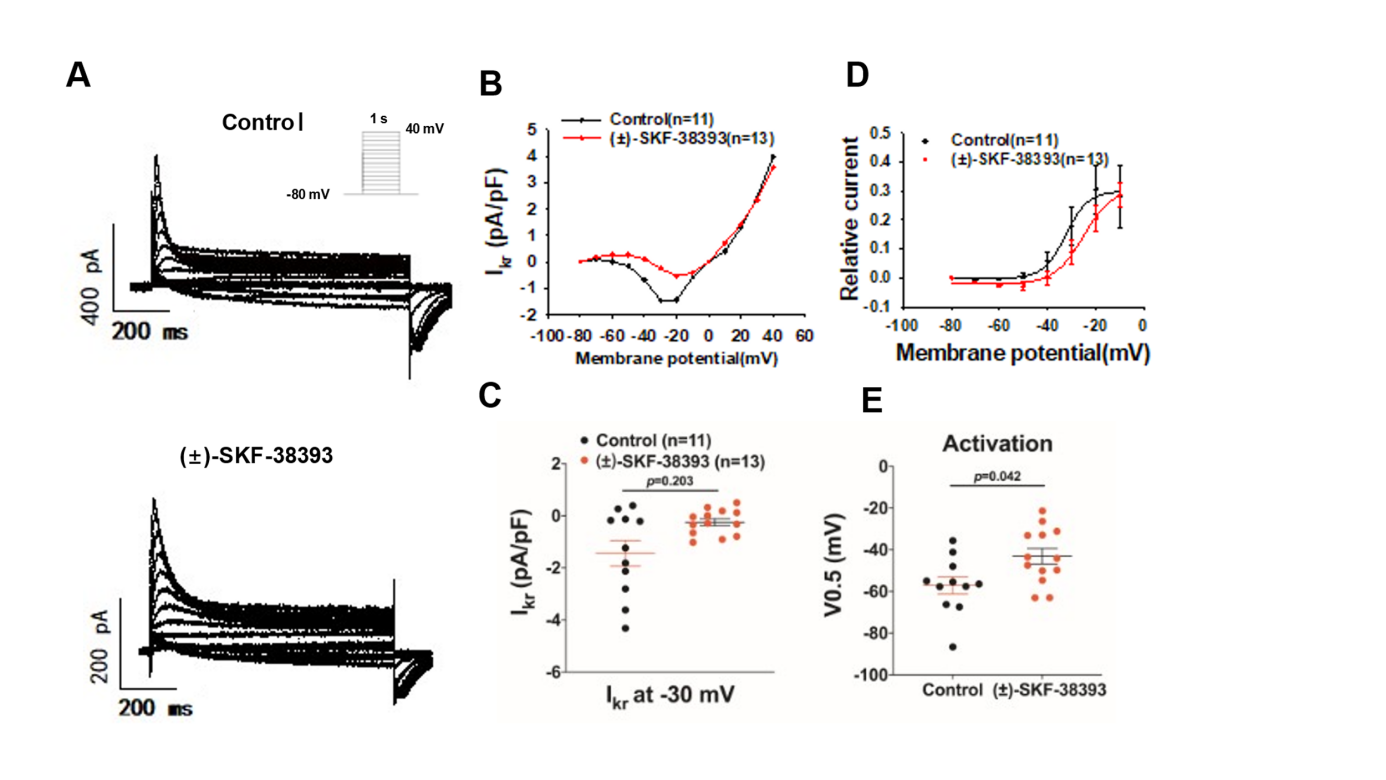


**Figure S6**

**
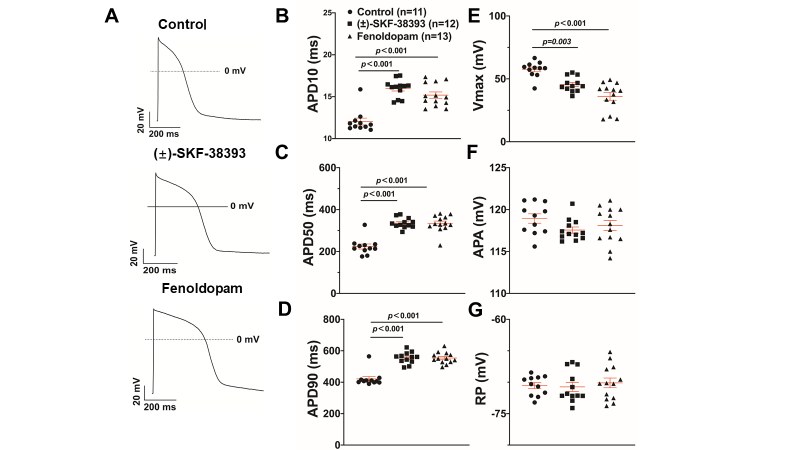
**

**Figure S7**

**
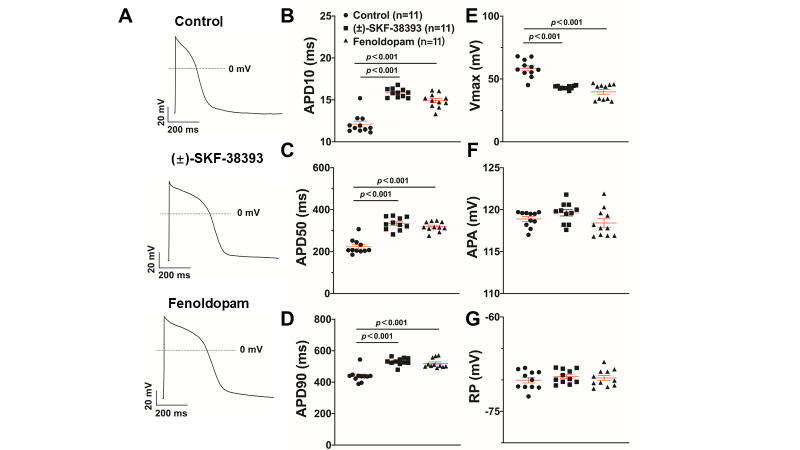
**
